# Origin, Expansion, and Divergence of ETHYLENE-INSENSITIVE 3 (EIN3)/EIN3-LIKE Transcription Factors During Streptophytes Evolution

**DOI:** 10.3389/fpls.2022.858477

**Published:** 2022-05-13

**Authors:** Kexin Mao, Minghui Zhang, Yadong Kong, Shanshan Dai, Yong Wang, Qingwei Meng, Nana Ma, Wei Lv

**Affiliations:** State Key Laboratory of Crop Biology, College of Life Sciences, Shandong Agricultural University, Tai’an, China

**Keywords:** ethylene signaling pathway, EIN3/EIL transcription factor, evolution, plant terrestrialization, phylogenetic analysis

## Abstract

The transition of plants to land required several regulatory adaptive mechanisms. Little is known about these mechanisms, but they no doubt involved the evolution of transcription factor (TF) families. ETHYLENE-INSENSITIVE 3 (EIN3)/EIN3-LIKE (EIL) transcription factors (TFs) are core components of the ethylene signaling pathway that play important roles in almost every aspect of plant development and environmental responses by regulating the transcription of numerous genes. However, the evolutionary history of EIN3/EIL TFs, which are present in a wide range of streptophytes, is still not clear. Here, to explore the evolution and functions of EIN3/EIL TFs, we performed phylogenetic analysis of these TFs and investigated their gene and protein structures as well as sequence features. Our results suggest that the EIN3/EIL TF family was already was already present in the ancestor of streptophyte algae. Phylogenetic analysis divided the EIN3/EIL TFs into three groups (Group A–C). Analysis of gene and protein structure revealed that most of the structural features of these TFs had already formed in ancient lineages. Further investigation suggested that all groups have undergone several duplication events related to whole-genome duplications in plants, generating multiple, functionally diverse gene copies. Therefore, as plants colonized terrestrial habitats and evolved key traits, the EIN3/EIL TF family expanded broadly *via* multiple duplication events, which could have promoted their fundamental neo- and sub-functionalization to help plants adapt to terrestrial life. Our findings shed light on the functional evolution of the EIN3/EIL TF family in the streptophytes.

## Introduction

The colonization and radiation of land plants were important milestones in the formation of the atmosphere and landscape on Earth. More than 450 million years ago, land plants evolved from a lineage of freshwater charophytes (Sanderson et al., 2004; Irisarri et al., 2021). The transition of plants from water to land was accompanied by morphological, physiological, and genetic changes to enhance adaption to conditions in the terrestrial environment, such as elevated CO_2_ concentrations, increased light intensity, drought, high and low temperatures, nutrient deficiency, and seasonal changes (Kenrick and Crane, 1997; Dahl et al., 2010; Delaux et al., 2012; Delwiche and Cooper, 2015). Despite their hundreds of millions of years of evolution, plants still cannot escape from hostile environments; therefore, plants have evolved sophisticated regulatory mechanisms and a series of innovations to respond multiple challenges posed by the external environment to ensure normal growth and development. Among the numerous regulatory mechanisms and innovations, the expansion of transcription factor (TF) families (de Mendoza et al., 2013; Holland, 2013; Catarino et al., 2016) have played significant roles in plant adaption to the terrestrial environment. The diversification of TF families in plant genomes suggests that TFs have played remarkable roles in plant adaptation to the changing environment, possibly through neo- and sub-functionalization (Zalewski et al., 2013; Hughes et al., 2014; Rensing, 2014; Moghe and Last, 2015).

The gaseous phytohormone ethylene (C_2_H_4_) has prominent effects on a broad spectrum of plant growth and defense processes (Qiao et al., 2012; Dubois et al., 2018). In the past decades, due to the identification of a series of key components using molecular and genetic approaches, the core ethylene signaling pathway has been well established (Chang et al., 1993; Hua et al., 1998; Sakai et al., 1998; Alonso et al., 1999). ETHYLENE-INSENSITIVE 3 (EIN3), a crucial TF in this pathway that can be degraded *via* the SCF (Skp-Cullin-F-box) E3 ligase complex with ETHYLENE INSENSITIVE3-BINDING F-BOX PROTEIN1/2 (EBF1/2; Kendrick and Chang, 2008), triggers ethylene responses by regulating the expression of *ETHYLENE PESPONSE FACTOR1/2* (*EPF1/2*).

In 1997, Chao et al. identified the *EIN3* gene and several related *EIN3-LIKE* (*EIL1*, *EIL2*, and *EIL3*) genes encoding positive regulators of the ethylene signaling pathway in *Arabidopsis* (Chao et al., 1997). Over the next 20 years, EIN3/EILs were identified in several plant species, such as tobacco (*Nicotiana tabacum*; Kosugi and Ohashi, 2000), tomato (*Solanum lycopersicum*; Tieman et al., 2001; Yokotani et al., 2003), and rice (*Oryza sativa*; Mao et al., 2006). Subsequent studies have shown that EIN3 and EILs are not only crucial downstream regulators in the ethylene signaling pathway, but they are also important factors in the crosstalk among various phytohormones (Yu et al., 2019). Therefore, an in-depth understanding of EIN3 function is crucial for clarifying the relationships between various signal transduction pathways during plant development and stress responses.

There are three clades of EIN3/EIL1 (A–C) in *Populus trichocarpa* and *Brassica napus* (Filiz et al., 2017; Li et al., 2019), each playing different roles in plant growth and development. Clade A contains EIN3 and EIL1, which are functionally homologous proteins involved in regulating ethylene-responsive gene expression (Chao et al., 1997; Solano et al., 1998; Alonso et al., 2003; An et al., 2010). EIL3 in clade B does not function in the ethylene signaling pathway but instead regulates the sulfur deficiency response (Wawrzyńska and Sirko, 2014, 2016). Clade C contains three proteins, EIL2, EIL4, and EIL5. Unlike EIN3 and EIL1, EIL2 plays only a minor role in plant responses to ethylene signals, as it partially complemented the *ein3* mutation (Chao et al., 1997). Nevertheless, little is known about the roles of EIL4 and EIL5 (Guo and Ecker, 2004). All EIN3/EILs are located in the nucleus and contain a DNA binding domain (DBD) that was shown to be required for DNA binding in *Arabidopsis* (Yamasaki et al., 2005) and tobacco (Kosugi and Ohashi, 2000). The N-termini of EIN3/EILs are highly conserved, including an acidic amino acid region, a proline-rich region, and five basic amino acid clusters (basic domain I-V, BD I-V; Chao et al., 1997). The structure of the N-terminal BD region is represented by the DBDs of EIN3/EILs, which recognize and bind directly to EIN3 binding site (EBS: “ATGTA”) in the promoter regions of downstream genes to activate or inhibit their expression (Chao et al., 1997). Song et al. revealed that amino acids 82–352 and 174–306 of EIN3 in *Arabidopsis* are the optimal and core DBDs (Song et al., 2015), respectively, and a 1.78 Å crystal structure of the core DBD of EIN3 [Protein Data Bank (PDB) accession number: 4ZDS] was identified containing BD III, BD IV, and the proline-rich region. These findings provide insights into the mechanistic details of key amino acid clusters involved in the DNA binding of EIN3. However, the C-terminal sequences are less conserved than the N-terminal sequences. For example, the poly-asparagine or poly-glutamine regions in the C-terminal sequences present in *Arabidopsis* (Lee and Kim, 2003) are absent in tobacco (Rieu et al., 2003). Therefore, analyzing the different motifs in the C-terminal sequences of these TFs could shed light on the evolutionary history of the EIN3/EIL family, such as gene duplication and gene loss events. However, there is still a lack of information regarding the roles of these motifs in the evolution of the EIN3/EIL family and their association with the functional roles of each class of EIN3/EIL TFs.

Although the three EIN3/EIL classes play different roles in numerous processes in plants (Filiz et al., 2017; Li et al., 2019), many of these processes do not exist in streptophyte algae. In addition, although the three EIN3/EIL classes were initially identified in land plants, some of these classes had their origins in streptophytes (Cheng et al., 2019). In this study, to better understand the evolution of EIN3/EIL TFs, we examined recently released databases spanning a wide range of plant taxa, including algae and land plant species, and identified EIN3/EIL families in streptophytes with the aim of elucidating the origins and expansion of the different EIN3/EIL families, variations in selection pressure, and functional divergence. We uncovered different aspects of the evolutionary history of the EIN3/EIL family, including gene duplication and gene loss events and the evolution of protein motifs in each family. Our results provide a theoretical basis for future functional and evolutionary research of EILs.

## Materials and Methods

### Identification, Nomenclature, and Characteristics of EIN3/EIL Family Proteins in Streptophytes

To analyze the diversity and evolution of EIN3/EIL proteins in streptophytes, all protein sequences available for 30 species were downloaded and used to construct a local protein database, including 5 streptophyte algae, 3 bryophytes, 1 lycophytes, 2 gymnosperms, 2 basal angiosperms, 10 eudicots, and 7 monocots: detailed information can be viewed in Supplementary Table S1. Two independent methods were employed to predict EIN3/EIL proteins in the entire protein dataset. First, HMMER 3.0 (Potter et al., 2018) was employed with a cutoff E-value of 1e-5 using PF04873, representing the newest HMM model for the EIN3/EIL domains downloaded from the Pfam database (Mistry et al., 2020)^1^ as a query model. Second, the Basic Local Alignment Search Tool (BLASTP) program (Camacho et al., 2009; Kong et al., 2013) was employed using the EIN3/EIL protein sequences (Supplementary Table S2 and Supplementary Data S1) downloaded in NCBI as the query sequences with a cutoff E-value of 1e-5. The accessions of these sequences were NP_188713, NP_180273, NP_001332194, NP_177514, NP_196574, and NP_201315. Finally, the two results were merged and examined for the presence of EIN3/EIL domains in the InterPro (Blum et al., 2020)^2^ and PROSITE (Sigrist et al., 2012; Ming et al., 2020)^3^ databases. The redundant sequences were removed manually based on the above results.

To match the names of the proteins with their function, all predicted EIN3/EIL proteins were named based on their evolutionary relationships. In the nomenclature system, the first word before the underscore represents the species name, the second word “EIL” after the underline indicates EIN3/EIL, and the number after “EIL” represents the classification in the phylogenetic tree. For example, the EIL1 protein in *Arabidopsis* was named Ath_EIL1.

The characteristics of all the predicted EIN3/EIL TFs were obtained. For example, the protein length, the molecular weight, and isoelectric point (pI) of the proteins were calculated *via* the ExPASy site (Wilkins et al., 1999).^4^ The gene length and exon number were calculated using customized perl programs (Supplementary Script S1).

### Multiple Sequence Alignment and Phylogenetic Analysis of EIN3/EIL Proteins

Multiple sequence alignment of predicted EIN3/EIL proteins was performed using Clustal W 2.0.3 (Thompson et al., 2002).^5^ The alignment logos of the conserved protein domains were generated with WebLogo (Crooks et al., 2004).^6^ To illustrate the evolutionary history of plant EIN3/EIL proteins, based on the results of multiple sequence alignment, PhyML (Guindon et al., 2010) was also used to set up Automatic model selection by SMS (Lefort et al., 2017) for the Maximum Likelihood (ML) phylogenetic evolutionary tree construction. IQ-TREE 2 (Minh et al., 2020) was used to construct a ML tree. And we rooted the trees on the branch separating the 4 *Chara braunii* sequences.

### Gene Structure and Protein Motif Analysis

Analysis of the exon/intron structures of all *EIN3*/*EIL* genes was performed using Gene Structure Display Server (GSDS) software (Hu et al., 2014)^7^ with the GFF version 3 file containing all *EIN3*/*EIL* gene models. Conserved motifs of EIN3/EIL proteins were identified using MEME suite (Bailey et al., 2015; Guo et al., 2017) with 20 motif numbers.

### Molecular Evolution Analysis

The ratios of the number of non-synonymous substitutions per non-synonymous site (Ka) to the number of synonymous substitutions per synonymous site (Ks) were used to calculate ω (Ka·Ks^−1^) values for the gene pairs of the target species (Hurst, 2002). As input files, the protein sequences and relative cDNA sequences must be consistent. The Simple Ka/Ks Calculator (NG) tool from TBtools (Nei and Gojobori, 1986; Wang et al., 2010; Chen et al., 2020) was used to perform calculations for each of the three A, B, and C groups and within their lower subclasses.

To examine the correspondence of *EIN3*/*EIL* genes among different species, we selected the dicotyledonous plants tomato (*Solanum lycopersicum*), soybean (*Glycine max*), *Arabidopsis thaliana*, and diploid cotton (*Gossypium raimondii*) and the monocotyledonous plants banana (*Musa acuminata*), rice (*Oryza sativa*), common wheat (*Triticum aestivum*), and maize (*Zea mays*) to analyze the synteny and collinearity of these genes among the genomes using MCScan software (Wang et al., 2012). In dating whole-genome duplication (WGD)/segmental duplication events, we used MCScanX to search for collinear *EIN3/EIL* gene pairs in the genomes.

## Results

### Identification and Phylogenetic Analysis of EIN3/EIL Proteins in Streptophytes

**Figure 1:**
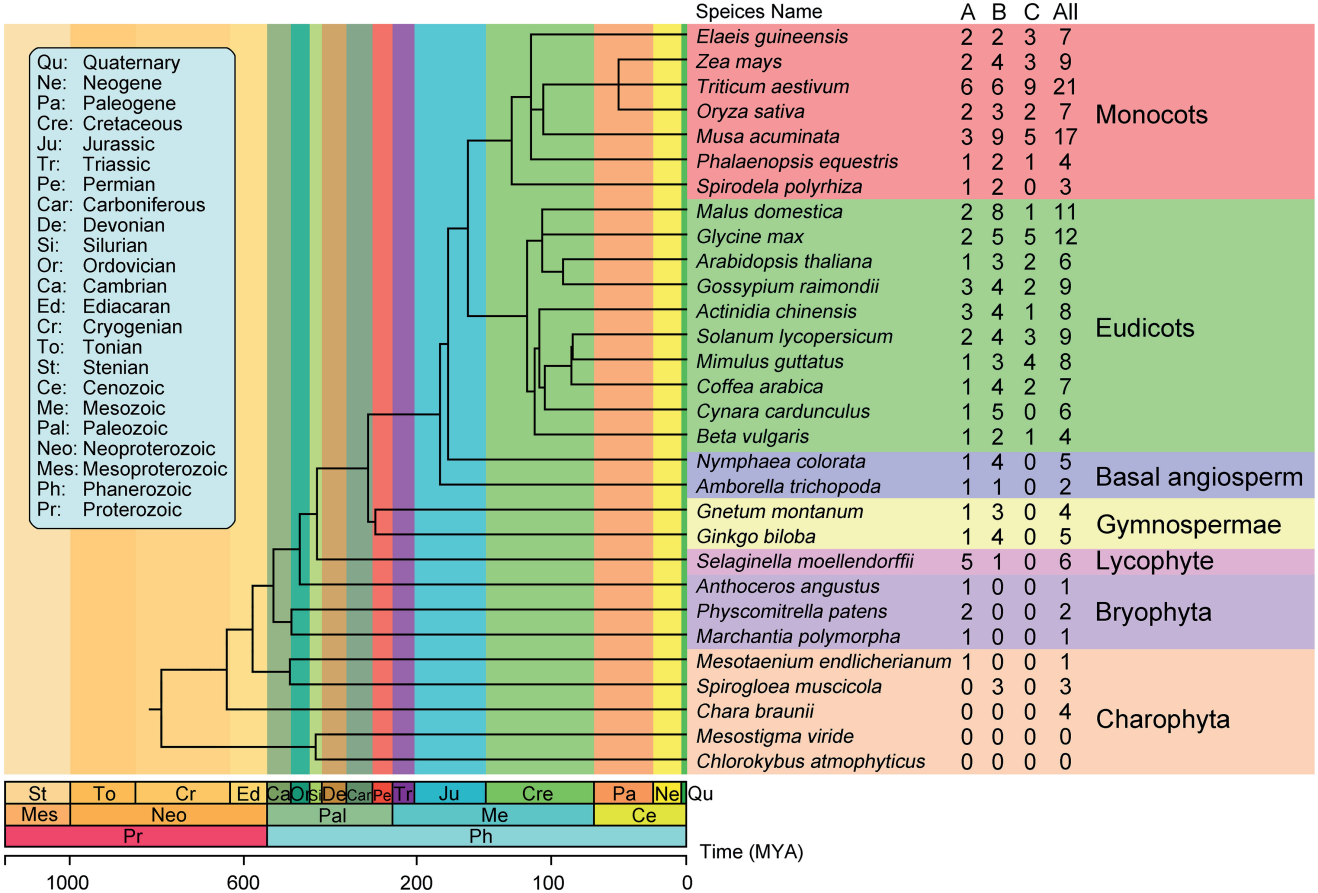
Phylogenetic relationships between the 30 plant species investigated in this study. The total number of EIL proteins and that of each groups identified in each plant genome is indicated on the right. The phylogenetic tree is modified from TIMETREE (http://timetree.org/).

To investigate the evolutionary history of plant EIN3/EIL proteins, we identified these proteins from 30 streptophytes whose genome are publicly available. The number of EIN3/EIL proteins varied among species. We identified 182 non-redundant EIN3/EIL proteins throughout streptophytes (Supplementary Table S3 and Supplementary Data S2). Two early-diverging streptophyte algae (*Chlorokybus atmophyticus*, *Mesostigma viride*; Wang et al., 2020) lack EIN3/EIL proteins, whereas *Spirogloea muscicola*, *Mesotaenium endlicherianum* and *Chara braunii* contain three, one, and four EIN3/EIL proteins, respectively. Bryophyta contain few EIN3/EIL proteins. For instance, only one such protein is present in *Marchantia polymorpha* and *Anthoceros angustus*. Lycophyta, Gymnosperm, and Angiosperm species contain more than two EIN3/EIL proteins, except for *Amborella trichopoda*, which contains two of these proteins (Figure 1).

**Figure 2:**
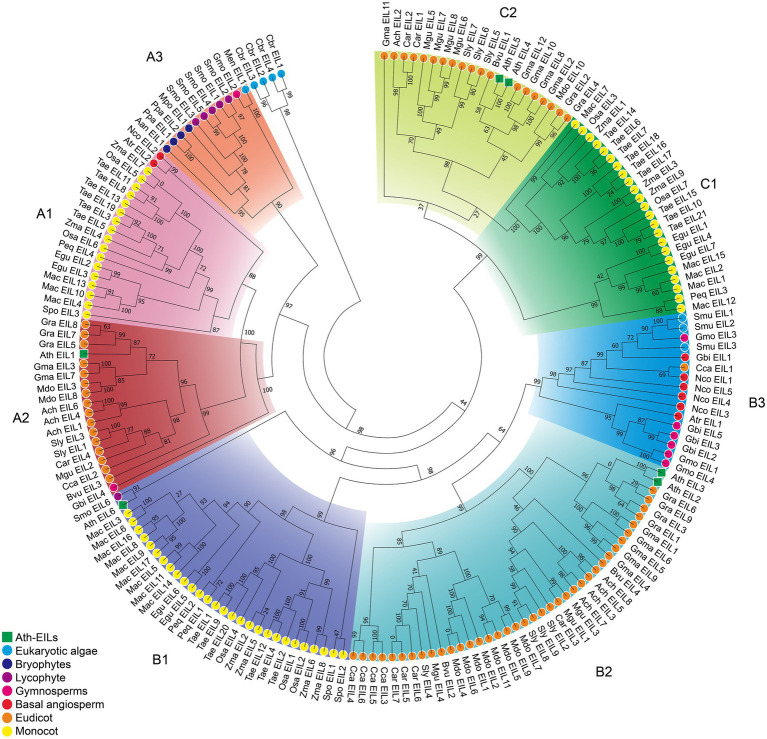
Phylogenetic analysis of 182 EIL proteins from 28 species. The phylogenetic tree of all sequences was constructed using PhyML by the Maximum Likelihood (ML) method. The root of the tree is the sequence of *Chara braunii*.

Based on phylogenetic analysis, the EIN3/EIL proteins were classified into three groups named A, B, and C, (Figure 2; Supplementary Figures S1–S3; Supplementary Data S3–S5), which is consistent with previous reports of EIN3/EIL proteins in angiosperms (Filiz et al., 2017; He et al., 2020; Jyoti et al., 2021). Moreover, as shown in Figure 2, group C EIN3/EIL proteins were only found in angiosperm and were divided into two subgroups: monocot (C1) and dicot (C2). By contrast, group A and B EIN3/EIL proteins were divided into monocot (A1 and B1) and dicot (A2 and B2), as well as streptophyte algae, bryophyte, lycophyte, gymnosperm, and basal angiosperm (A3 and B3). Notably, no streptophyte algae, bryophyte, lycophyte, gymnosperm, or basal angiosperm contained group C EIN3/EIL proteins, suggesting that these proteins appeared later in evolution. Most streptophyte algae, Bryophytes, and Lycophyte contained only group A EIN3/EIL proteins, except for *Spirogloea muscicola* and *Chara braunii*, indicating that ancient EIN3/EIL proteins existed prior to the separation of streptophyte algae and land plants. This notion was confirmed by the identification of EIN3/EIL proteins in streptophyte algae, such as *Klebsormidium nitens* and *Chara braunii* (Nishiyama et al., 2018).

### Characteristics of EIN3/EIL Gene and Protein in Subfamilies

*EIN3*/*EIL* genes ranged from 843 bp (*Smo_EIL3*) to 17,414 bp (*Egu_EIL4*), with an average of 2,670 bp (Supplementary Table S3 and Supplementary Figure S4A). The greatest intra-group bipolar variation in gene length was found in group C, and the least such variation was found in group B. The median gene length for group A was approximately 2,500 bp. The gene length of group B was less variable than others, and median of the subgroups in group B was similar to the lower 1/4 locus (Q1), except the subgroup B2. Most sequences in group C were shorter than others, but there were some long gene sequences (more than 1 k bp).

The proteins encoded by the *EIN3*/*EIL* genes ranged from 185 (Cca_EIL5) to 896 amino acid (Cbr_EIL3), with an average of 566 amino acid (Supplementary Table S3 and Supplementary Figure S4B). The predicted molecular weights of the proteins ranged from 21.011 kD (Cca_EIL5) to 95.070 kD (Men_EIL1), with an average molecular weight of 63.198 kD (Supplementary Table S3 and Supplementary Figure S4C). The medians of group A1-A3 seem to be similar, but the data for group A2 and A3 were more variable than A1. The members of group C had the shortest protein lengths and the lowest molecular weights among groups.

The isoelectric points (pIs) of these proteins ranged from 4.59 (Mac_EIL17) to 10.27 (Nco_EIL5; Supplementary Table S3 and Supplementary Figure S4D). The pIs were similar among subgroups, but those of subgroup A3 were more variable than other subgroups. Among these proteins, 93.4% were acidic, and the remaining 6.6% were basic proteins.

Subgroup A2, A3, and B3 contained more exon than the other subgroups (Supplementary Table S3 and Supplementary Figure S4E).

### Common Conserved Domain Compositions and Genomic Analysis of EIN3/EIL Proteins in Plants

MEME analysis of EIL protein motifs not only demonstrated the evolutionary conservation of the DNA binding domain (DBD), but it also identified protein motifs specific to different subgroups, laying a foundation for in-depth study of their functions. Song et al. determined that amino acid 82–352 and 174–306 of EIN3 in *Arabidopsis* act as the optimal and core DBDs and resolved the 1.78 Å crystal structure of the core DBD [Protein Data Bank (PDB) accession number: 4ZDS], which contains BD III, BD IV, and a proline-rich region (Song et al., 2015). The molecular structure shows that the EIL DBD consists of six α-helices. As shown by multiple sequence alignment (Supplementary Figures S5–S12), all subgroups possessed the DBD. These results indicate that all these EIL members retained the ability to bind DNA during the evolutionary process and that the diversity of their regulation probably was due to sub- or neo-functionalization. These results provide insights into the mechanistic details of key amino acid motifs involved in the DNA binding of EIN3.

**Figure 3:**
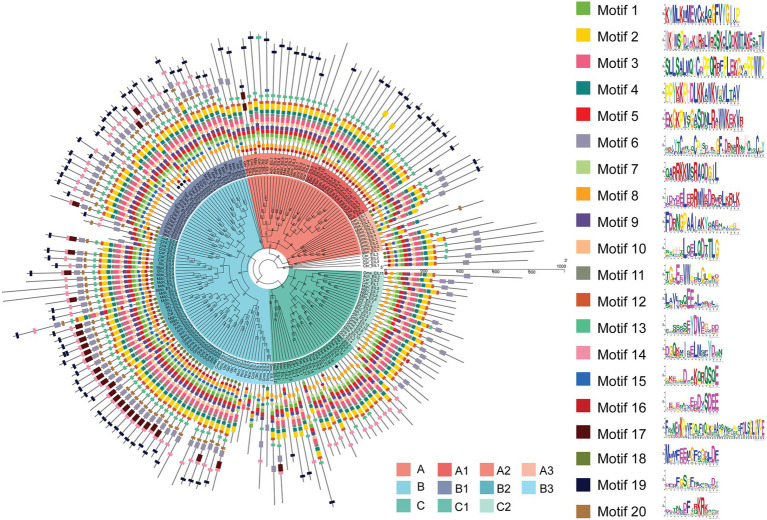
Phylogenetic relationship and conserved motifs of all EILs. A total of 182 EIN3/EIL proteins from 28 species were selected to construct the phylogenetic tree using PhyML by ML method. Conserved motifs of the EIN3/EIL proteins were obtained using the MEME software.

**Figure 4:**
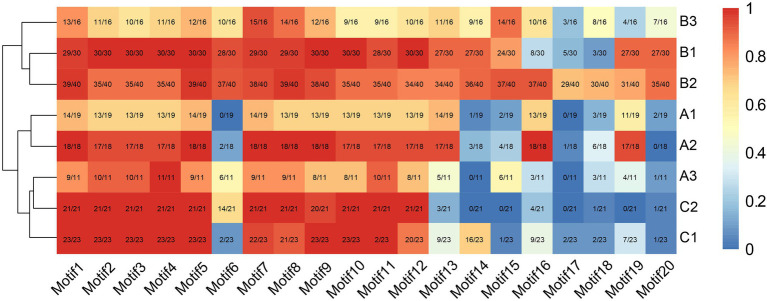
The distribution of each motif structure in different subgroups. The red indicates more occurrences, blue indicates fewer occurrences.

Consistent with the results of multiple sequence alignment, motif analysis showed that the B3 subgroup was the least conservative, and about half the members did not contain motifs 2 and 4, that was the BD III and BD IV domains, for example Nco_EIL1, Cca_EIL1, Smu_EIL3, and Gmu_EIL3. And the EIL proteins of *Cynara cardunculus* (Cca_EIL3-6 in subgroup B2 and Cca EIL_2 in subgroup A2) did not have BD III and BD IV. All other subgroups possessed the conserved BD I-IV domains and six α-helices of the DBD, which were mainly distributed in motifs 2, 3, 4, 7, 8, 10, 11, and 12. Most sequences in subgroups A1, A2, B1, B2, C1, and C2 contained motifs 2, 3, 4, 7, 8, 10, 11, and 12, while only some of the sequences of subgroups A3 and B3 contained the above motifs. Therefore, the DBD of EIL originated from streptophyte algae, and the DBD is closely related to the transcriptional regulatory function of these proteins (Figures 3, 4). By contrast, motifs 2, 3, 4, 7, 8, 10, 11, and 12 are highly conserved in angiosperms, with all EIL sequences except *Cynara cardunculus* containing the above motifs, suggesting that in angiosperms, including both dicotyledons and monocotyledons, EIL proteins contain a DBD consistent with that of *Arabidopsis* EIN3 and exhibit a conserved structure (Song et al., 2015). During evolution, the DBD of EIL, the most basic structural and functional element of these proteins, was highly conserved among different species. However, during long-term evolution, TFs underwent sub- or neo-functionalization through WGD or tandem duplication, resulting in functional diversity (Sémon and Wolfe, 2007). In turn, the variation in motifs among subfamilies inevitably led to sequence diversity. We further analyzed the motifs of different subgroups of EIL proteins and determined that different subgroup sequences had their own specific motifs. In particular, angiosperms in subgroups A1, A2, B1, and B2 contained the specific motifs 19 at their C-termini, and most members of the B3 subgroup, including gymnosperms and basal angiosperms, also possessed these motifs. Motif 20 was mostly present in group B, especially B1 and B2. Motif 20 contained BD V, a domain that regulates the DNA binding capacity of EIN3 in *Arabidopsis*, which may be closely related to the regulation of ethylene-responsive genes by EIL proteins (Lee et al., 2006). In addition, motif 16 was relatively conserved in subgroup A1, A2, B2, and B3. However, this motif was mostly absent in other families, especially group C, perhaps due to the late acquisition of sequences in group C during evolution due to partial fragment deletion.

**Figure 5:**
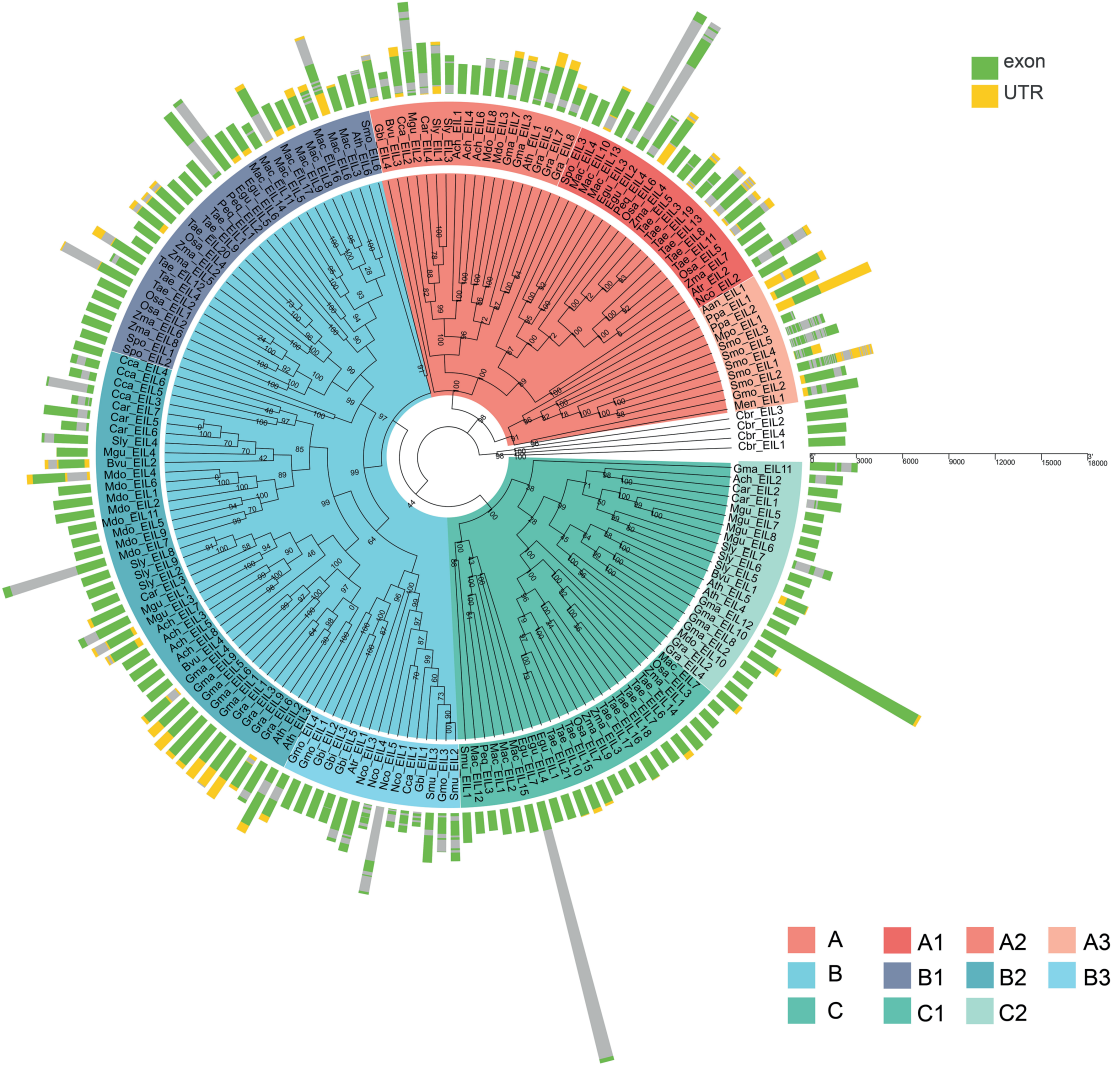
The exon-intron structure of 182 EIN3/EILs genes. Green boxes indicate untranslated 5′- and 3′-regions, yellow boxes indicate UTR, and the grey lines indicate introns.

To further explore the structural diversity of the *EIL* genes, we analyzed the exons/introns of each *EIL* gene and their corresponding genomic DNA sequences. The number of exons in most algae or early land plants was highly variable, ranging from 1 to 11 in subgroups, such as A3 and B3. Most *EIL* genes of the monocotyledons and dicotyledons from the late evolutionary period were composed of one or two exons, such as those of subgroups A1, A2, B1, B2, C1, and C2, perhaps due to intron loss during evolution (Figure 5; Supplementary Figure S3E).

### The Role of Selection Pressure in the Expansion and Diversity of the *EIN3* Family

**Figure 6:**
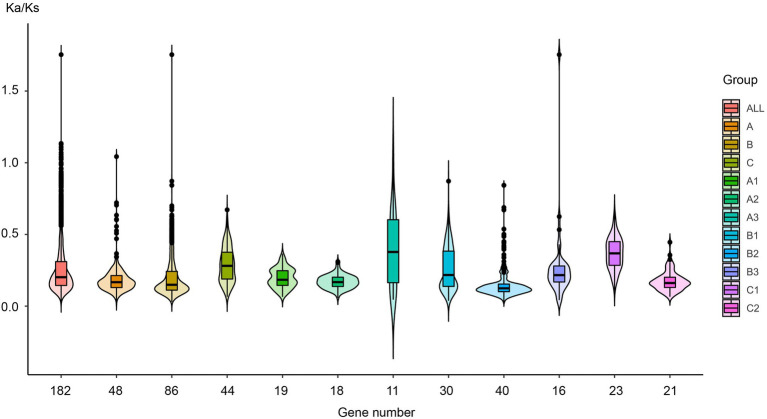
The Ka/Ks value distribution in different plant lineages. Each EIL was compared with the other EILs in the same plant lineage one by one, and the Ka/Ks value was estimated for each compared pair, and the colors are labeled with the different subgroups.

Natural selection leads to the functional diversity of genes, such as neo-functionalization, sub-functionalization, de-functionalization, and so on (Flagel and Wendel, 2009; Wendel et al., 2016). To evaluate the effects of sequence diversification on the degree of functional conservation of these genes, we calculated the ratio of non-synonymous substitutions (Ka) to synonymous substitutions (Ks) for each pair of gene sequences (*ω* = Ka/Ks) and analyzed their selection pressure. Figure 6 and Supplementary Data S6 show that the *ω* value was basically less than 0.25 in almost all species and that these sequences had undergone purifying selection to ensure the stability of their biological functions.

For the entire EIL family, the ω value was approximately 0.25, indicating strong purifying selection, leading to the assumption that its functions were quite conserved during evolution. All members of groups A, B, and C had been subjected to purifying selection during evolution, but their selection pressures were different. The 86 sequences of group B had been subjected to the strongest purifying selection, indicating that they had the highest functional conservation. Both EIN3 and EIL1 are core TFs in the ethylene signaling pathway belonging to group B. This further demonstrates the immutable importance of EIL TFs in the ethylene signaling pathway. Group C sequences were only detected in dicotyledons and monocotyledons, whose ancestors probably originated at a late stage of plant evolution. Group C sequences had relatively high ω values, especially in the monocotyledons of subgroup C1, which originated most recently. Due to the decreased selection pressure, these sequences may produce functional diversity. From the perspective of different subgroups, the selection pressure obviously changed during plant evolution. The ω value (median values and dispersion) of subgroups A3, and B3 from streptophyte algae to gymnosperms is larger than that of subgroups A1, A2, B1, B2, and C2 of angiosperms, suggesting that during the early stage of plant evolution, due to gene duplication and sequence diversity, many genes underwent functional diversity under relatively low selection pressure.

### Synteny and Collinearity Analysis of *EIL* Genes in Dicotyledonous and Monocotyledonous Plants

**Figure 7:**
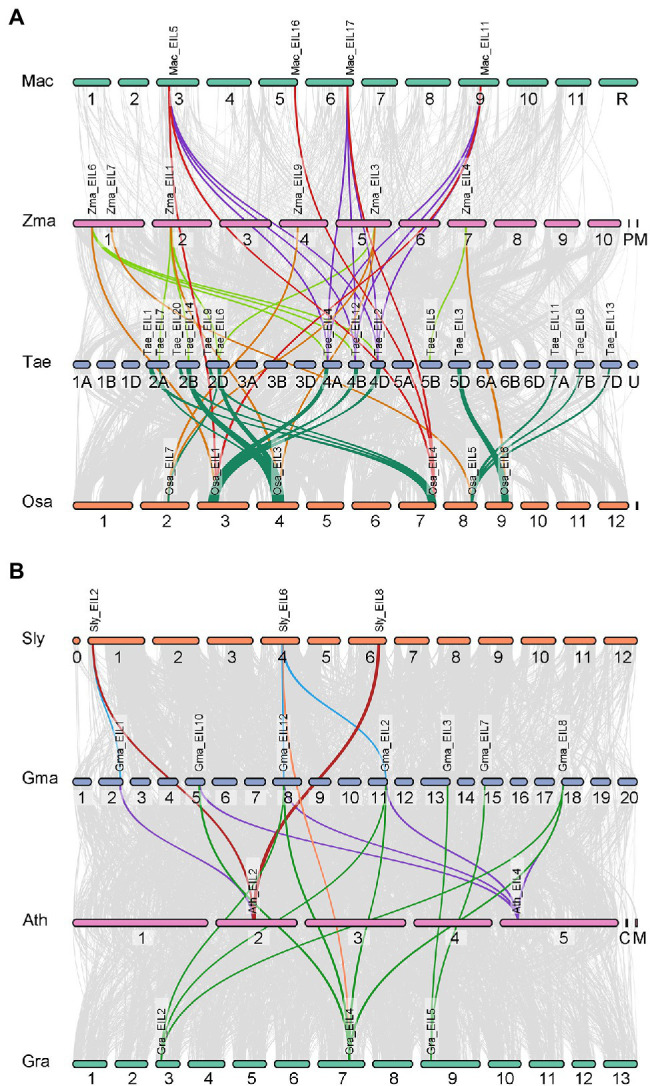
Synteny analysis of EINs/EIL genes between four dicotyledons and four monocotyledons. Gray lines in the background indicate the collinear blocks within two plant genomes, while the colorful lines highlight the syntenic EIL gene pairs. **(A)** Synteny analysis of EINs/EIL genes between four monocotyledons. And the species names with “Mac,” “Zma,” “Tae,” “Osa” indicate *Musa acuminata*, *Zea mays*, *Triticum aestivum*, and *Oryza sativa*, respectively. **(B)** Synteny analysis of EINs/EIL genes between four dicotyledons. And the species names with “Sly,” “Gma,” “Ath,” “Gra” indicate *Solanum lycopersicum*, *Glycine max*, *Arabidopsis thaliana*, and *Gossypium raimondii*, respectively.

Gene duplication plays an important role in plant evolution. The number of TFs is usually amplified through gene duplication events, resulting in functional diversity. Since the emergence of angiosperms, plants have undergone three major large-scale genome-wide replication events, providing numerous gene sources for the growth and development of angiosperms and their adaptation to the environment. To further explore the role of gene duplication in the expansion and variation of the *EIL* gene family in angiosperms, we mapped collinear genes to the genomes of four monocotyledons (*Musa acuminata*, *Oryza sativa*, *Triticum aestivum*, *Zea mays*) and four dicotyledons (*Solanum lycopersicum*, *Glycine max*, *Arabidopsis thaliana*, *Gossypium raimondii*; Figure 7). Significantly more interspecific collinear gene pairs were present in monocotyledons (43 pairs) than dicotyledons (20 pairs). There are two possible reasons for this. First, the monocotyledons used for analysis include wheat, which itself contains three sets of collinear genes. Second, monocotyledons originated later than dicotyledons and produced relatively few gene loss events. Although collinear gene pairs significantly differed in monocotyledonous vs. dicotyledonous plants, the distribution of collinear gene pairs was unequal among different groups in all plant species examined. Group A contained nine collinear gene pairs, including seven from monocotyledons (16.3%) and two from dicotyledons (10%). Group B contained 28 collinear gene pairs, including 24 from monocotyledons (55.8%) and four from dicotyledons (20%). The distribution of collinear gene pairs in group C was opposite that of group B, with 12 monocotyledonous pairs (27.9%) and 14 dicotyledonous pairs (70%). These results indicate that there were fewer interspecific collinear gene pairs in group A from angiosperms, likely due to their earlier origination and their numerous gene loss events. We suggest that the collinear gene pairs in groups B and C of angiosperms had the same origin, underwent genome-wide duplication events, and were retained. Therefore, genes from groups B and C show functional diversity, providing a strong material basis for the evolution of plants.

## Discussion

Ethylene regulates the ripening of respiratory climacteric fruits. This leads to a wide range of physiological and morphological responses in plants, such as inhibited cell expansion, the promotion of leaf and flower senescence, and the induction of fruit ripening and abscission (An et al., 2018). EIN3 and EIL proteins are positive downstream regulators of the ethylene signaling pathway.

Here, we identified 182 EIN3/EIL family members from 30 plant species whose genomes were publicly available. *Chlorokybus atmophyticus* and *Mesostigma viride* lack EIN3/EIL protein. The earliest species possessing an EIL protein was *Chara braunii*, which belongs to the Charophyceae and contains four EIL protein. Three and one EIL proteins were identified in *Spirogloea muscicola* and *Mesotaenium endlicherianum*, respectively. Based on phylogenetic analysis, three EIN3/EIL protein groups were identified and named A, B, and C, which is consistent with previously reported EIN3/EIL proteins in angiosperms.

In the present study, we rooted the trees on the branch separating the 4 *Chara braunii* sequences. Notably, most A3 and B3 subgroup members had a specific exon structure, and lacked the core domain, suggesting that the EILs in the subgroups had lost some functions or acquired new functions. However, there is currently no evidence to support this speculation. No group C member was identified in non-angiosperms. Perhaps these sequences emerged after the separation of gymnosperms and angiosperms. Alternatively, perhaps ancestors of these sequences were lost in non-angiosperms.

To better understand the characteristics of EIN3/EIL proteins in different plants, we further analyzed their sequence features. Multiple sequence alignment of proteins from different subgroups showed that group A, B, and C proteins contained conserved BD I-IV and proline-rich domains in algae and dicotyledons. The DBD was shown to be required for DNA binding in *Arabidopsis* (Yamasaki et al., 2005) and tobacco (Kosugi and Ohashi, 2000). We identified highly conserved N-termini in the EIN3/EILs, including an acidic amino acid region, a proline-rich region, and five basic amino acid clusters (BD I-V; Chao et al., 1997). Notably, the BD V structure appeared only in group B, which may be related to the specificity of its function. AtEIN3 (Ath_EIL3) and AtEIL1 (Ath_EIL2) of *Arabidopsis* belong to the B2 subgroup (Solano et al., 1998), and tomato LeEIL1 (Sly_EIL8), LeEIL2 (Sly_EIL2), and LeEIL3 (Sly_EIL4; Tieman et al., 2001) as well as rice OsEIL1 (Osa_EIL2; Mao et al., 2006) also belong to the B group. These observations suggest that group B EILs play a crucial role in the ethylene signaling pathway.

The diversity of terrestrial environments poses a major challenge to plant survival, and polyploidy has emerged during the evolution of many plants. There was only one to four *EIL* genes in streptophyte algae, but six in *Arabidopsis*, indicating that *EIL* genes expanded during plant evolution. Interestingly, approximately 400 million years ago (MYA), the EIL family expanded dramatically from gymnosperms during plant evolution. The average number of EIL genes ranged from 4.5 in gymnosperms to 8 in eudicots and 9.7 in monocots (Figure 1 and Supplementary Table S1). Correspondingly, the ratio of EIL number to total protein number in each species ranged from 0.0133% in gymnosperm to 0.0184% in eudicots and 0.0209% in monocots, showing a significant expansion of the EIL family from gymnosperms to angiosperms during plant evolution. This result is consistent with the finding that the first round of ancestral WGD occurred 319 MYA (Chaw et al., 2004; Jiao et al., 2011) and that several rounds of lineage-specific WGD subsequently occurred (Bowers et al., 2003).

Several mechanisms contribute to genome size variation in eukaryotes from yeast to organisms with more complex genomes, such as vertebrates. Two of the most important mechanisms are WGDs due to either autopolyploidy or hybridization and the accumulation of transposable elements. WGDs have been extensively studied in plants since the discovery of this process in the *Arabidopsis* genome in 2000 (Blanc et al., 2000). Interestingly, in some cases, polyploid genomes are able to return to disomy *via* a diploidization process, such as gene loss, mutation, and sub-functionalization (Kuzmin et al., 2020). These processes have important consequences for gene copies. WGD is thought to participate in the evolution and adaptation of organisms (Soltis et al., 2009). Several methodologies have been developed to detect paleo-polyploidy in genomic sequences, especially in plants, and to explore its relevance (Qiao et al., 2019). Gene families can contain large subfamilies due to events, such as segmental duplication, tandem duplication, or conversion events (Cannon et al., 2004; Kong et al., 2007). Tandem and segmental duplications are thought to be the two main causes of gene duplication in plants (Cannon et al., 2004). Duplication events can promote the emergence of new genes, which can help increase the diversity of gene function and effectively improve the ability of plants to adapt to different environments (Flagel and Wendel, 2009). Interestingly, the basal angiosperm *Amborella trichopoda* contains only two EILs, as there was no evidence for recent genome replication in a particular strain (*Amborella Genome* Project). However, we determined that both the number of *EIL* genes and the proportion of EILs to all proteins were quite variable in streptophyte algae and non-angiosperms that arose approximately 400 MYA. This result is inconsistent with previous studies showing that gene families expanded in dendritic plants to help them conquer terrestrial habitats by increasing tolerance to environmental stress (Rensing et al., 2008). This discrepancy may be due to the small number of genomic datasets available for early land plants. On the other hand, it may result from the loss of genes in specific species after WGD. For example, in *Physcomitrium patens*, a fusion event occurred during haploidization between two ancestral WGD events, resulting in the loss of EIL genes on chromosomes 1 and 2. Therefore, there are only two *EILs* on chromosomes 7 and 11 in *Physcomitrium patens*, which shares an ancestral chromosome with chromosomes 1 and 2 (Lang et al., 2018). These results suggest that *EIL* genes expanded during the evolution of gymnosperms due to WGD events. Most *EIL* genes were retained after the WGD event in angiosperms, which was more pronounced in monocots. Given that the *EIL* gene family began to expand in angiosperms, which coincided with a WGD event 319 MYA, we hypothesize that the evolution of the EIL family laid the material basis for the subsequent emergence of angiosperms.

## Conclusion

We identified 182 EIL genes in 30 plant species using bioinformatics approaches. Phylogenetic analysis divided the EIN3/EIL TFs into three groups (Group A-C). Group A and B EILs first appeared in the common ancestors of all green plants, whereas group C EILs arose concomitantly with the emergence of angiosperms. Our results demonstrate that the EIL family was already present in the ancestor of streptophyte algae and that its expansion was accompanied by important developmental processes and environmental diversity. In angiosperms, due to the occurrence of WGDs, the number of EIL proteins has increased significantly, perhaps resulting in neo- or sub-functionalization of genes, thereby allowing plants to adapt to the ever-changing environment. These findings shed new light on the functions and evolutionary history of plant EILs.

## Data Availability Statement

The original contributions presented in the study are included in the article/Supplementary Material, further inquiries can be directed to the corresponding authors.

## Author Contributions

KM, MZ, YK, and SD carried out the public genome data collection. KM and MZ performed the data analyses. YW and QM contributed to the study design. KM, NM, and WL wrote the manuscript. All authors were involved in the revision of the manuscript and approved the final manuscript.

## Funding

This work was supported by the National Natural Science Foundation of China (31870277, 31900208, and 31870239).

## Conflict of Interest

The authors declare that the research was conducted in the absence of any commercial or financial relationships that could be construed as a potential conflict of interest.

## Publisher’s Note

All claims expressed in this article are solely those of the authors and do not necessarily represent those of their affiliated organizations, or those of the publisher, the editors and the reviewers. Any product that may be evaluated in this article, or claim that may be made by its manufacturer, is not guaranteed or endorsed by the publisher.

## Footnotes

^1^ http://pfam.xfam.org/

^2^ http://www.ebi.ac.uk/interpro/

^3^ http://prosite.expasy.org/

^4^ http://web.expasy.org/protparam/

^5^ http://www.ebi.ac.uk/clustalw/

^6^ http://weblogo.threeplusone.com/

^7^ http://gsds.cbi.pku.edu.cn/

## Supplementary Material

Supplementary Figure S1 Phylogenetic analysis of 182 EIL proteins from 28 species. The phylogenetic tree of all sequences was constructed using IQ-TREE 2 by the Maximum Likelihood (ML) method.

Supplementary Figure S2 The phylogenetic tree was used to construct the maximum likelihood (ML) trees with the programs of PhyML with branch lengths and support values.

Supplementary Figure S3 The phylogenetic tree was used to construct the maximum likelihood (ML) trees with the programs of IQ-TREE 2 with branch lengths and support values.

Supplementary Figure S4 The gene length **(A)**, protein length **(B)**, protein molecular weight **(C)**, protein isoelectric point **(D)**, and exon number value **(E)** distribution in different plant lineages. Each EIL was compared with the other EILs in the same plant lineage one by one, and the characteristic of EIL was estimated for each compared pair, and the colors are labeled with the different subgroups.

Supplementary Figure S5 Sequence alignment of A1 subgroup EIL proteins. The sequence alignment was performed using ClustalX.

Supplementary Figure S6 Sequence alignment of A2 subgroup EIL proteins.

Supplementary Figure S7 Sequence alignment of A3 subgroup EIL proteins.

Supplementary Figure S8 Sequence alignment of B1 subgroup EIL proteins.

Supplementary Figure S9 Sequence alignment of B2 subgroup EIL proteins.

Supplementary Figure S10 Sequence alignment of B3 subgroup EIL proteins.

Supplementary Figure S11 Sequence alignment of C1 subgroup EIL proteins.

Supplementary Figure S12 Sequence alignment of C2 subgroup EIL proteins.

Supplementary Data S1 The sequences file of the EIN3/EIL proteins downloaded in NCBI (Supplementary Table S2).

Supplementary Data S2 The sequences file of the EIN3/EIL proteins (Supplementary Table S3).

Supplementary Data S3 The newick/nexus tree files of the ML tree with the programs of PhyML.

Supplementary Data S4 The newick/nexus tree files of the ML tree with the programs of IQ-TREE 2.

Supplementary Data S5 The alignments used to infer the phylogenetic trees.

Supplementary Data S6 The aligned nucleotide datasets used for Ka/Ks analyses.

Supplementary Script S1 The perl script used for the gene length, and exon numbers.
