# Supplementary material for: Origin, Expansion, and Divergence of ETHYLENE-INSENSITIVE 3 (EIN3)/EIN3-LIKE Transcription Factors During Streptophytes Evolution
Source: Front Plant Sci. 2022 May 13;13:858477. doi: 10.3389/fpls.2022.858477 (PMC9136324; doi:10.3389/fpls.2022.858477)
Supplement: Supplementary Figure S1 — Phylogenetic analysis of 182 EIL proteins from 28 species. The phylogenetic tree of all sequences was constructed using IQ-TREE 2 by the Maximum Likelihood (ML) method. [file Data_Sheet_1.ZIP › Table S3.docx]

Table S3. The list of the EIN3/EIL proteins

| Abbreviation | Protein length | Protein MW | Protein PI | Gene length | Exon number | ID in database | Database | Species |
| --- | --- | --- | --- | --- | --- | --- | --- | --- |
| Smu_EIL1 | 665 | 70340.75 | 6.93 | 3118 | 5 | SM000004S15101 | figshare | *Spirogloea muscicola* |
| Smu_EIL2 | 451 | 46527.84 | 6.31 | 2525 | 4 | SM000013S26441 |  |  |
| Smu_EIL3 | 202 | 22145.84 | 5.78 | 955 | 3 | SM004979S17083 |  |  |
| Men_EIL1 | 866 | 95070.33 | 5.68 | 3602 | 3 | ME000112S10988 | figshare | *Mesotaenium endlicherianum* |
| Cbr_EIL1 | 796 | 86671.93 | 6.85 | 2388 | 1 | g17647 | NCBI | *Chara braunii* |
| Cbr_EIL2 | 892 | 94292.15 | 6.64 | 2676 | 1 | g30137 |  |  |
| Cbr_EIL3 | 896 | 94618.26 | 6.51 | 2688 | 1 | g57605 |  |  |
| Cbr_EIL4 | 833 | 89261.1 | 6.39 | 2499 | 1 | g6607 |  |  |
| Mpo_EIL1 | 616 | 68369.37 | 5.37 | 6548 | 1 | Mapoly0088s0024 | JGI | *Marchantia polymorpha* |
| Ppa_EIL1 | 680 | 73453.94 | 5.19 | 4258 | 1 | Pp3c7_9970V3 | JGI | *Physcomitrium patens* |
| Ppa_EIL2 | 678 | 74112.53 | 5.33 | 3317 | 1 | Pp3c11_15260V3 |  |  |
| Aan_EIL1 | 643 | 70902.55 | 5.47 | 1929 | 1 | AANG001212 | dryad | *Anthoceros angustus* |
| Smo_EIL1 | 276 | 30695.28 | 9.93 | 1782 | 5 | 449238 | JGI | *Selaginella moellendorffii* |
| Smo_EIL2 | 574 | 65333.82 | 9.81 | 4984 | 11 | 448939 |  |  |
| Smo_EIL3 | 250 | 28316.9 | 9.86 | 843 | 2 | 112063 |  |  |
| Smo_EIL4 | 608 | 69100.61 | 9.59 | 3206 | 11 | 419761 |  |  |
| Smo_EIL5 | 416 | 48314.7 | 8.65 | 2572 | 11 | 419770 |  |  |
| Smo_EIL6 | 649 | 71943.73 | 5.81 | 1950 | 1 | 16064 |  |  |
| Gbi_EIL1 | 388 | 44415.39 | 5.02 | 1421 | 3 | Gb_03629 | PLAZA | *Ginkgo biloba* |
| Gbi_EIL2 | 668 | 75143.19 | 5.58 | 2007 | 1 | Gb_15228 |  |  |
| Gbi_EIL3 | 643 | 72430.58 | 5.38 | 1932 | 1 | Gb_03292 |  |  |
| Gbi_EIL4 | 667 | 73972.7 | 5.33 | 2200 | 3 | Gb_36016 |  |  |
| Gbi_EIL5 | 637 | 71325.98 | 5.56 | 1914 | 1 | Gb_08309 |  |  |
| Gmo_EIL1 | 296 | 33459.44 | 8.81 | 3369 | 1 | TnS000824405t01 | PLAZA | *Gnetum montanum* |
| Gmo_EIL2 | 632 | 71242.38 | 5.48 | 2995 | 1 | TnS000650041t01 |  |  |
| Gmo_EIL3 | 574 | 62879.08 | 5.47 | 3236 | 2 | TnS000182403t06 |  |  |
| Gmo_EIL4 | 666 | 74332.64 | 6.25 | 4633 | 1 | TnS000331101t01 |  |  |
| Atr_EIL1 | 638 | 71361.01 | 5.69 | 1866 | 1 | ATR0068G063 | JGI | *Amborella trichopoda* |
| Atr_EIL2 | 622 | 69268.58 | 5.51 | 1943 | 2 | ATR0605G094 |  |  |
| Nco_EIL1 | 328 | 36436.89 | 5.83 | 5754 | 4 | NC10G0233770 | NCBI | *Nymphaea colorata* |
| Nco_EIL2 | 468 | 52406.63 | 5.04 | 4238 | 4 | NC6G0268800 |  |  |
| Nco_EIL3 | 612 | 68180.12 | 5.74 | 2934 | 1 | NC1G0065150 |  |  |
| Nco_EIL4 | 623 | 69857.04 | 5.49 | 3369 | 2 | NC9G0200390 |  |  |
| Nco_EIL5 | 342 | 39975.6 | 10.27 | 1635 | 5 | NC8G0300100 |  |  |
| Bvu_EIL1 | 473 | 53002.1 | 6.26 | 1944 | 1 | Bv4_071090_wzrs | Ensembl Plant | *Beta vulgaris* |
| Bvu_EIL2 | 698 | 77321.52 | 5.5 | 4088 | 1 | Bv4_096120_faoj |  |  |
| Bvu_EIL3 | 611 | 68175.99 | 5.29 | 3004 | 2 | Bv8_197040_ozas |  |  |
| Bvu_EIL4 | 619 | 70578.74 | 5.54 | 2977 | 1 | Bv9_207000_nzcd |  |  |
| Cca_EIL1 | 219 | 24876.31 | 9.09 | 1208 | 3 | KVI08603 | Ensembl Plant | *Cynara cardunculus* |
| Cca_EIL2 | 420 | 47115.29 | 4.98 | 1733 | 2 | KVH90820 |  |  |
| Cca_EIL3 | 260 | 29237.38 | 4.71 | 1321 | 3 | KVI03067 |  |  |
| Cca_EIL4 | 444 | 50596.22 | 4.92 | 1881 | 2 | KVI08478 |  |  |
| Cca_EIL5 | 185 | 21011.49 | 6.04 | 3094 | 2 | KVI03139 |  |  |
| Cca_EIL6 | 417 | 45724.83 | 5.07 | 1767 | 2 | KVH92019 |  |  |
| Car_EIL1 | 470 | 53610.81 | 5.59 | 2248 | 3 | XP_027075715 | JGI | *Coffea arabica* |
| Car_EIL2 | 463 | 52849.61 | 4.97 | 1904 | 2 | XP_027078201 |  |  |
| Car_EIL3 | 634 | 71844.79 | 5.48 | 3595 | 1 | XP_027081715 |  |  |
| Car_EIL4 | 680 | 77028.17 | 5.85 | 6001 | 3 | XP_027095299 |  |  |
| Car_EIL5 | 311 | 35565 | 8.79 | 1787 | 1 | XP_027103132 |  |  |
| Car_EIL6 | 608 | 68528.8 | 5.3 | 2992 | 1 | XP_027107316 |  |  |
| Car_EIL7 | 608 | 68333.56 | 5.3 | 2992 | 1 | XP_027114277 |  |  |
| Mgu_EIL1 | 582 | 65790.86 | 5.46 | 3100 | 1 | Migut.N02421 | JGI | *Mimulus guttatus* |
| Mgu_EIL2 | 646 | 71551.48 | 5.38 | 3215 | 2 | Migut.N02294 |  |  |
| Mgu_EIL3 | 598 | 67536.93 | 5.86 | 2620 | 1 | Migut.D00719 |  |  |
| Mgu_EIL4 | 562 | 63223.36 | 5.07 | 2890 | 1 | Migut.B01164 |  |  |
| Mgu_EIL5 | 411 | 46412.48 | 5.26 | 1233 | 1 | Migut.H02377 |  |  |
| Mgu_EIL6 | 418 | 47571.59 | 4.8 | 1254 | 1 | Migut.H02375 |  |  |
| Mgu_EIL7 | 417 | 46936.89 | 4.89 | 1251 | 1 | Migut.H02373 |  |  |
| Mgu_EIL8 | 417 | 47398.23 | 4.81 | 1251 | 1 | Migut.H02376 |  |  |
| Sly_EIL1 | 661 | 73805.42 | 6.17 | 3317 | 2 | Solyc01g006650 | JGI | *Solanum lycopersicum* |
| Sly_EIL2 | 615 | 69136.31 | 5.32 | 2494 | 1 | Solyc01g009170 |  |  |
| Sly_EIL3 | 676 | 76072.63 | 6 | 3334 | 2 | Solyc01g014480 |  |  |
| Sly_EIL4 | 602 | 67879.47 | 5.33 | 2007 | 1 | Solyc01g096810 |  |  |
| Sly_EIL5 | 435 | 50289.91 | 5.79 | 1305 | 1 | Solyc03g096630 |  |  |
| Sly_EIL6 | 504 | 58361.61 | 5.02 | 1512 | 1 | Solyc04g054840 |  |  |
| Sly_EIL7 | 367 | 42228.82 | 8.77 | 2646 | 3 | Solyc05g051875 |  |  |
| Sly_EIL8 | 611 | 69481.48 | 5.86 | 1833 | 1 | Solyc06g073720 |  |  |
| Sly_EIL9 | 606 | 68665.77 | 5.68 | 1818 | 1 | Solyc06g073730 |  |  |
| Ach_EIL1 | 608 | 68487.97 | 5.84 | 2459 | 3 | Achn123771 | Ensembl Plant | *Actinidia chinensis* |
| Ach_EIL2 | 492 | 56035.09 | 5.31 | 2175 | 2 | Achn171621 |  |  |
| Ach_EIL3 | 627 | 70813.81 | 5.82 | 1881 | 1 | Achn017871 |  |  |
| Ach_EIL4 | 638 | 71628.44 | 5.65 | 1914 | 1 | Achn018171 |  |  |
| Ach_EIL5 | 619 | 70129.42 | 5.49 | 1857 | 1 | Achn018431 |  |  |
| Ach_EIL6 | 626 | 70238.79 | 5.22 | 1878 | 1 | Achn346641 |  |  |
| Ach_EIL7 | 621 | 70269.17 | 5.46 | 1863 | 1 | Achn346881 |  |  |
| Ach_EIL8 | 599 | 67892.75 | 5.86 | 1797 | 1 | Achn347321 |  |  |
| Gra_EIL1 | 615 | 69590.44 | 5.48 | 3418 | 1 | KJB20248 | JGI | *Gossypium raimondii* |
| Gra_EIL2 | 504 | 57401.84 | 5.02 | 1515 | 1 | KJB18267 |  |  |
| Gra_EIL3 | 614 | 69745.77 | 5.58 | 3743 | 1 | KJB26156 |  |  |
| Gra_EIL4 | 482 | 55568.49 | 5.28 | 1791 | 1 | KJB44228 |  |  |
| Gra_EIL5 | 601 | 67798.16 | 5.68 | 3656 | 2 | KJB55330 |  |  |
| Gra_EIL6 | 612 | 69398.36 | 5.49 | 3770 | 1 | KJB56070 |  |  |
| Gra_EIL7 | 686 | 77445.27 | 5.97 | 3404 | 2 | KJB66132 |  |  |
| Gra_EIL8 | 363 | 40882.56 | 9.27 | 2088 | 2 | KJB83499 |  |  |
| Gra_EIL9 | 594 | 66816.37 | 5.12 | 3517 | 1 | KJB83827 |  |  |
| Ath_EIL1 | 567 | 64041.53 | 5.28 | 2484 | 2 | AT1G73730 | JGI | *Arabidopsis thaliana* |
| Ath_EIL2 | 584 | 66495.44 | 5.83 | 2541 | 1 | AT2G27050 |  |  |
| Ath_EIL3 | 628 | 71421.41 | 5.62 | 2921 | 1 | AT3G20770 |  |  |
| Ath_EIL4 | 471 | 53954.14 | 5.3 | 1416 | 1 | AT5G10120 |  |  |
| Ath_EIL5 | 557 | 63689.59 | 4.77 | 1722 | 1 | AT5G65100 |  |  |
| Ath_EIL6 | 518 | 59185.71 | 5.75 | 1674 | 1 | AT5G21120 |  |  |
| Gma_EIL1 | 614 | 69589.49 | 5.49 | 3521 | 1 | KRH73465 | JGI | *Glycine max* |
| Gma_EIL2 | 398 | 45263.15 | 5.08 | 1326 | 1 | KRH31276 |  |  |
| Gma_EIL3 | 591 | 66052.93 | 5.77 | 2968 | 2 | KRH23176 |  |  |
| Gma_EIL4 | 621 | 70451.49 | 5.33 | 3219 | 1 | KRH18695 |  |  |
| Gma_EIL5 | 618 | 70088.24 | 5.51 | 3404 | 1 | KRH18696 |  |  |
| Gma_EIL6 | 610 | 69010.99 | 5.45 | 3448 | 1 | KRH14682 |  |  |
| Gma_EIL7 | 590 | 66129.09 | 5.75 | 3113 | 2 | KRH10163 |  |  |
| Gma_EIL8 | 464 | 52506.33 | 4.88 | 2315 | 1 | KRG97597 |  |  |
| Gma_EIL9 | 624 | 70651.75 | 5.51 | 3517 | 1 | KRG89855 |  |  |
| Gma_EIL10 | 462 | 53133.77 | 5.1 | 1389 | 1 | KRH59382 |  |  |
| Gma_EIL11 | 766 | 84847.37 | 5.82 | 3094 | 3 | KRH56267 |  |  |
| Gma_EIL12 | 453 | 52092.6 | 5.03 | 11646 | 1 | KRH43217 |  |  |
| Mdo_EIL1 | 841 | 92654.05 | 4.84 | 2526 | 1 | HF17629-RA | JGI | *Malus domestica* |
| Mdo_EIL2 | 610 | 69093.88 | 4.94 | 1833 | 1 | HF06633-RA |  |  |
| Mdo_EIL3 | 604 | 67499.59 | 5.54 | 2380 | 2 | HF39780-RA |  |  |
| Mdo_EIL4 | 704 | 78427.73 | 5.09 | 2115 | 1 | HF14170-RA |  |  |
| Mdo_EIL5 | 605 | 68250.98 | 5.71 | 1818 | 1 | HF14173-RA |  |  |
| Mdo_EIL6 | 841 | 92654.05 | 4.84 | 2526 | 1 | HF16534-RA |  |  |
| Mdo_EIL7 | 625 | 70655.33 | 5.25 | 1878 | 1 | HF34524-RA |  |  |
| Mdo_EIL8 | 606 | 67615.58 | 5.95 | 2402 | 2 | HF43797-RA |  |  |
| Mdo_EIL9 | 822 | 93242.55 | 5.44 | 6914 | 2 | HF17013-RA |  |  |
| Mdo_EIL10 | 434 | 48872.98 | 5.63 | 1305 | 1 | HF35406-RA |  |  |
| Mdo_EIL11 | 600 | 67932.62 | 5.66 | 1803 | 1 | HF06632-RA |  |  |
| Spo_EIL1 | 575 | 62418.51 | 5.96 | 1725 | 1 | Spipo0G0106000 | JGI | *Spirodela polyrhiza* |
| Spo_EIL2 | 634 | 69519.92 | 5.48 | 1902 | 1 | Spipo3G0015900 |  |  |
| Spo_EIL3 | 598 | 66076.6 | 5.76 | 1794 | 1 | Spipo5G0016000 |  |  |
| Peq_EIL1 | 574 | 64725.48 | 5.18 | 1722 | 1 | PEQU_00346 | PLAZA | *Phalaenopsis equestris* |
| Peq_EIL2 | 643 | 71909.14 | 5.48 | 1929 | 1 | PEQU_10689 |  |  |
| Peq_EIL3 | 474 | 53506.35 | 5.61 | 1422 | 1 | PEQU_16294 |  |  |
| Peq_EIL4 | 591 | 66285.82 | 5.59 | 1751 | 2 | PEQU_19283 |  |  |
| Mac_EIL1 | 435 | 48791.92 | 5.39 | 1305 | 1 | MAC01G1130 | JGI | *Musa acuminata* |
| Mac_EIL2 | 552 | 62207.17 | 5.16 | 1656 | 1 | MAC06G2120 |  |  |
| Mac_EIL3 | 519 | 58584.13 | 6.22 | 5270 | 3 | MAC06G2602 |  |  |
| Mac_EIL4 | 572 | 63596.5 | 6.78 | 1716 | 1 | MAC07G2728 |  |  |
| Mac_EIL5 | 530 | 59953.56 | 6.19 | 1731 | 2 | MAC07G3240 |  |  |
| Mac_EIL6 | 523 | 58757.39 | 6.18 | 2323 | 4 | MAC08G0811 |  |  |
| Mac_EIL7 | 338 | 38244.69 | 9.17 | 1014 | 1 | MAC08G1489 |  |  |
| Mac_EIL8 | 593 | 66239.19 | 6.88 | 2013 | 1 | MAC10G2036 |  |  |
| Mac_EIL9 | 605 | 68334.8 | 5.31 | 2189 | 1 | MAC12G2339 |  |  |
| Mac_EIL10 | 611 | 67662.6 | 5.43 | 3039 | 1 | MAC02G0928 |  |  |
| Mac_EIL11 | 623 | 70346.22 | 5.99 | 2220 | 1 | MAC02G2236 |  |  |
| Mac_EIL12 | 656 | 72630.45 | 6.25 | 1968 | 1 | MAC03G0083 |  |  |
| Mac_EIL13 | 617 | 68511.74 | 6.11 | 1851 | 1 | MAC04G1218 |  |  |
| Mac_EIL14 | 635 | 71355.09 | 5.68 | 2239 | 1 | MAC04G2776 |  |  |
| Mac_EIL15 | 548 | 61850.8 | 5.05 | 1644 | 1 | MAC04G3320 |  |  |
| Mac_EIL16 | 628 | 70240.78 | 5.24 | 2442 | 1 | MAC05G1855 |  |  |
| Mac_EIL17 | 693 | 77828.05 | 4.59 | 4071 | 2 | MAC06G1189 |  |  |
| Osa_EIL1 | 644 | 71034.63 | 4.95 | 1932 | 1 | LOC_Os03g20780 | JGI | *Oryza sativa* |
| Osa_EIL2 | 641 | 70813.42 | 5.07 | 1923 | 1 | LOC_Os03g20790 |  |  |
| Osa_EIL3 | 446 | 48324.77 | 5.02 | 1338 | 1 | LOC_Os04g38400 |  |  |
| Osa_EIL4 | 594 | 65146.9 | 5.3 | 2811 | 1 | LOC_Os07g48630 |  |  |
| Osa_EIL5 | 620 | 69161.48 | 6.43 | 3283 | 1 | LOC_Os08g39830 |  |  |
| Osa_EIL6 | 612 | 68283.69 | 5.58 | 3843 | 1 | LOC_Os09g31400 |  |  |
| Osa_EIL7 | 505 | 54438.32 | 5.1 | 1684 | 1 | LOC_Os02g36510 |  |  |
| Tae_EIL1 | 618 | 67299.78 | 5.66 | 1857 | 1 | TraesCS2A02G099900 | JGI | *Triticum aestivum* |
| Tae_EIL2 | 651 | 71846.45 | 5.07 | 5072 | 1 | TraesCS4D02G177000 |  |  |
| Tae_EIL3 | 612 | 68385.88 | 5.9 | 2684 | 1 | TraesCS5D02G273600 |  |  |
| Tae_EIL4 | 547 | 60518.04 | 5.41 | 1956 | 2 | TraesCS4A02G129400 |  |  |
| Tae_EIL5 | 582 | 64695.35 | 5.5 | 1836 | 2 | TraesCS5B02G265400 |  |  |
| Tae_EIL6 | 465 | 51478.58 | 5.07 | 1795 | 1 | TraesCS2D02G320800 |  |  |
| Tae_EIL7 | 466 | 51445.36 | 4.91 | 1401 | 1 | TraesCS2A02G342500 |  |  |
| Tae_EIL8 | 613 | 67719.1 | 5.55 | 3221 | 1 | TraesCS7B02G145400 |  |  |
| Tae_EIL9 | 620 | 67628.99 | 5.66 | 2630 | 1 | TraesCS2D02G099400 |  |  |
| Tae_EIL10 | 458 | 50815.16 | 5.48 | 1740 | 1 | TraesCS3D02G390000 |  |  |
| Tae_EIL11 | 614 | 67855.22 | 5.58 | 2877 | 1 | TraesCS7A02G246100 |  |  |
| Tae_EIL12 | 650 | 71930.59 | 5.07 | 3798 | 1 | TraesCS4B02G175100 |  |  |
| Tae_EIL13 | 614 | 67924.35 | 5.68 | 3220 | 1 | TraesCS7D02G244600 |  |  |
| Tae_EIL14 | 466 | 51518.54 | 5.12 | 1711 | 1 | TraesCS2B02G340200 |  |  |
| Tae_EIL15 | 437 | 48463.56 | 5.21 | 1843 | 1 | TraesCS3B02G428000 |  |  |
| Tae_EIL16 | 458 | 50790.22 | 5.54 | 1620 | 1 | TraesCS6B02G376700 |  |  |
| Tae_EIL17 | 452 | 50035.4 | 6.07 | 1359 | 1 | TraesCSU02G080900 |  |  |
| Tae_EIL18 | 458 | 50665.13 | 5.34 | 1377 | 1 | TraesCS6D02G326600 |  |  |
| Tae_EIL19 | 583 | 64856.68 | 5.37 | 1839 | 2 | TraesCS5A02G265700 |  |  |
| Tae_EIL20 | 619 | 67217.65 | 5.65 | 2542 | 1 | TraesCS2B02G116900 |  |  |
| Tae_EIL21 | 452 | 50245.62 | 5.61 | 1639 | 1 | TraesCS3A02G396000 |  |  |
| Zma_EIL1 | 533 | 58665.27 | 5.28 | 1599 | 1 | Zm00001d003451 | JGI | *Zea mays* |
| Zma_EIL2 | 596 | 63812.04 | 6.34 | 1788 | 1 | Zm00001d007188 |  |  |
| Zma_EIL3 | 430 | 47557.53 | 5.1 | 1290 | 1 | Zm00001d016924 |  |  |
| Zma_EIL4 | 610 | 68145.63 | 5.92 | 1830 | 1 | Zm00001d020939 |  |  |
| Zma_EIL5 | 455 | 48326.16 | 5.47 | 1365 | 1 | Zm00001d022530 |  |  |
| Zma_EIL6 | 648 | 72034.94 | 5.05 | 1944 | 1 | Zm00001d028974 |  |  |
| Zma_EIL7 | 633 | 69195.76 | 5.75 | 1899 | 1 | Zm00001d031445 |  |  |
| Zma_EIL8 | 643 | 71390.01 | 5.1 | 1929 | 1 | Zm00001d047563 |  |  |
| Zma_EIL9 | 442 | 48306.46 | 4.92 | 1326 | 1 | Zm00001d050861 |  |  |
| Egu_EIL1 | 525 | 58374.84 | 5.86 | 1593 | 1 | XP_010923033 | PLAZA | *Elaeis guineensis* |
| Egu_EIL2 | 605 | 68072.93 | 5.56 | 9370 | 1 | XP_010924320 |  |  |
| Egu_EIL3 | 607 | 67389.54 | 5.48 | 9495 | 1 | XP_010926635 |  |  |
| Egu_EIL4 | 723 | 80610.78 | 5.85 | 17414 | 2 | XP_010931518 |  |  |
| Egu_EIL5 | 636 | 70777.67 | 5.14 | 5173 | 1 | XP_010943685 |  |  |
| Egu_EIL6 | 649 | 72320.5 | 5.04 | 5444 | 1 | XP_019701622 |  |  |
| Egu_EIL7 | 613 | 67563.92 | 5.75 | 1842 | 1 | XP_029120761 |  |  |
