# Supplementary material for: Origin, Expansion, and Divergence of ETHYLENE-INSENSITIVE 3 (EIN3)/EIN3-LIKE Transcription Factors During Streptophytes Evolution
Source: Front Plant Sci. 2022 May 13;13:858477. doi: 10.3389/fpls.2022.858477 (PMC9136324; doi:10.3389/fpls.2022.858477)
Supplement: Supplementary Figure S1 — Phylogenetic analysis of 182 EIL proteins from 28 species. The phylogenetic tree of all sequences was constructed using IQ-TREE 2 by the Maximum Likelihood (ML) method. [file Data_Sheet_1.ZIP › Table S1.docx]

Table S1. The list of the 30 plant species

| Group | Species | Abbreviation | Genes number | EIN3/EIL number |
| --- | --- | --- | --- | --- |
| Streptophyte algae | *Chlorokybus atmophyticus* | Cat | 9,300 | 0 |
|  | *Mesostigma viride* | Mvi | 9,300 | 0 |
|  | *Spirogloea muscicola* | Smu | 27,137 | 3 |
|  | *Mesotaenium endlicherianum* | Men | 11,080 | 1 |
|  | *Chara braunii* | Cbr | 35,885 | 4 |
| Bryophyte | *Marchantia polymorpha* | Mpo | 24,674 | 1 |
|  | *Physcomitrium patens* | Ppa | 87,533 | 2 |
|  | *Anthoceros angustus* | Aan | 14,629 | 1 |
| Lycophyte | *Selaginella moellendorffii* | Smo | 22,285 | 6 |
| Gymnosperm | *Ginkgo biloba* | Gbi | 41,309 | 5 |
|  | *Gnetum montanum* | Gmo | 27,491 | 4 |
| Basal angiosperm | *Amborella trichopoda* | Atr | 26,846 | 2 |
|  | *Nymphaea colorata* | Nco | 28,438 | 5 |
| Eudicot | *Beta vulgaris* | Bvu | 29,088 | 4 |
|  | *Cynara cardunculus* | Cca | 26,505 | 6 |
|  | *Coffea arabica* | Car | 67,222 | 7 |
|  | *Mimulus guttatus* | Mgu | 33,573 | 8 |
|  | *Solanum lycopersicum* | Sly | 34,075 | 9 |
|  | *Actinidia chinensis* | Ach | 39,038 | 8 |
|  | *Gossypium raimondii* | Gra | 78,371 | 9 |
|  | *Arabidopsis thaliana* | Ath | 35,386 | 6 |
|  | *Glycine max* | Gma | 88,412 | 12 |
|  | *Malus domestica* | Mdo | 44,677 | 11 |
| Monocot | *Spirodela polyrhiza* | Spo | 19,623 | 3 |
|  | *Phalaenopsis equestris* | Peq | 29,415 | 4 |
|  | *Musa acuminata* | Mac | 36,528 | 17 |
|  | *Oryza sativa* | Osa | 42,189 | 7 |
|  | *Triticum aestivum* | Tae | 133,346 | 21 |
|  | *Zea mays* | Zma | 39,498 | 9 |
|  | *Elaeis guineensis* | Egu | 43,551 | 7 |
