# Supplementary material for: Origin, Expansion, and Divergence of ETHYLENE-INSENSITIVE 3 (EIN3)/EIN3-LIKE Transcription Factors During Streptophytes Evolution
Source: Front Plant Sci. 2022 May 13;13:858477. doi: 10.3389/fpls.2022.858477 (PMC9136324; doi:10.3389/fpls.2022.858477)
Supplement: Supplementary Figure S1 — Phylogenetic analysis of 182 EIL proteins from 28 species. The phylogenetic tree of all sequences was constructed using IQ-TREE 2 by the Maximum Likelihood (ML) method. [file Data_Sheet_1.ZIP › Table S2.docx]

Table S2. The list of the EIN3/EIL proteins downloaded in NCBI

| NCBI number | Name | NCBI name | Species |
| --- | --- | --- | --- |
| NP_188713 | EIN3 | Ethylene insensitive 3 family protein | *Arabidopsis thaliana* |
| NP_180273 | EIL1 | ETHYLENE-INSENSITIVE3-like 1 | *Arabidopsis thaliana* |
| NP_001332194 | EIL2 | ETHYLENE-INSENSITIVE3-like 2 | *Arabidopsis thaliana* |
| NP_177514 | EIL3 | ETHYLENE-INSENSITIVE3-like 3 | *Arabidopsis thaliana* |
| NP_196574 | EIL4 | Ethylene insensitive 3 family protein | *Arabidopsis thaliana* |
| NP_201315 | EIL5 | Ethylene insensitive 3 family protein | *Arabidopsis thaliana* |
