# Supplementary figures and images for: Origin, Expansion, and Divergence of ETHYLENE-INSENSITIVE 3 (EIN3)/EIN3-LIKE Transcription Factors During Streptophytes Evolution
Source: Front Plant Sci. 2022 May 13;13:858477. doi: 10.3389/fpls.2022.858477 (PMC9136324; doi:10.3389/fpls.2022.858477)

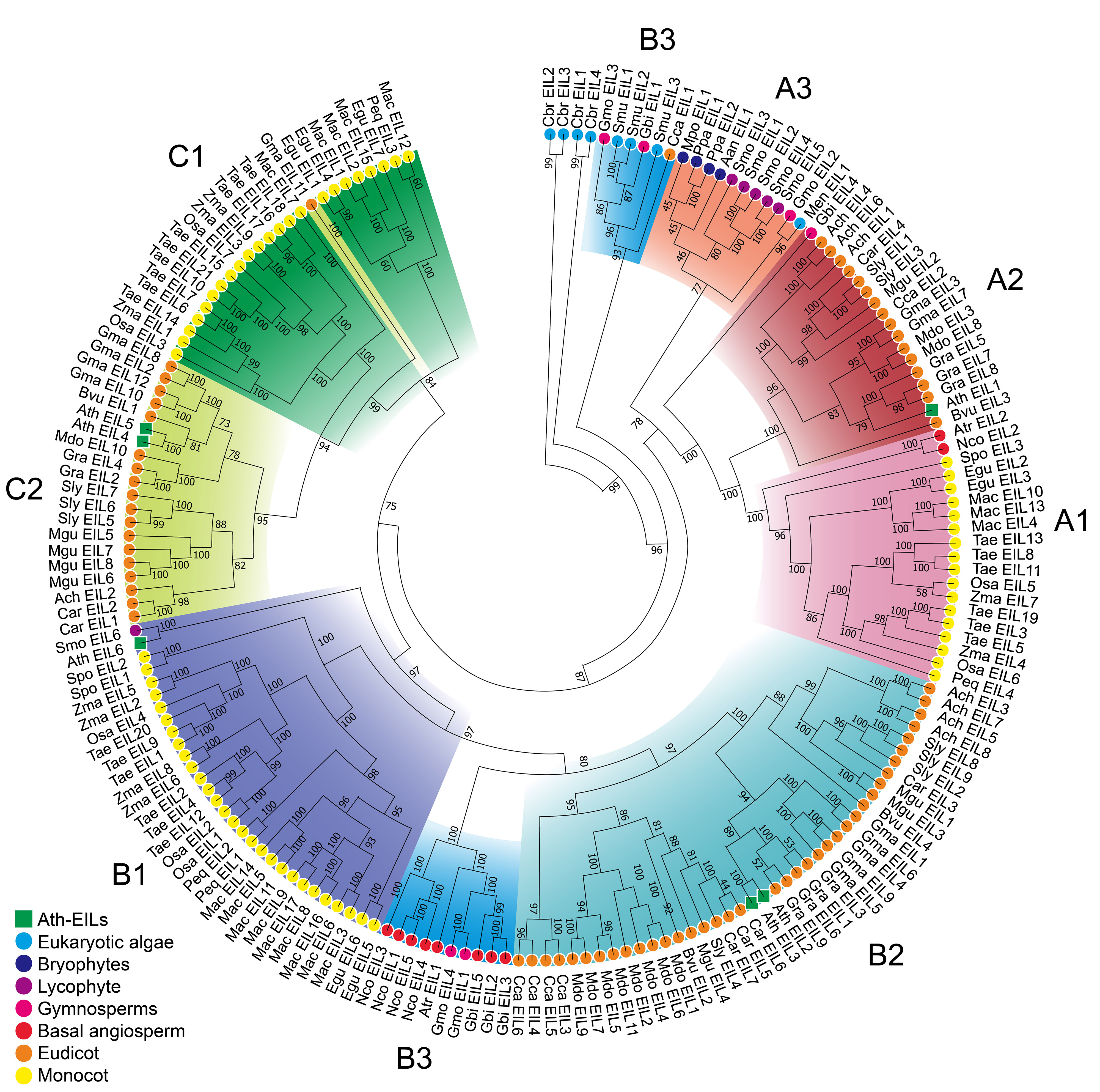

Supplement: Supplementary Figure S1 — Phylogenetic analysis of 182 EIL proteins from 28 species. The phylogenetic tree of all sequences was constructed using IQ-TREE 2 by the Maximum Likelihood (ML) method. [file Data_Sheet_1.ZIP › Figure S1.jpg]

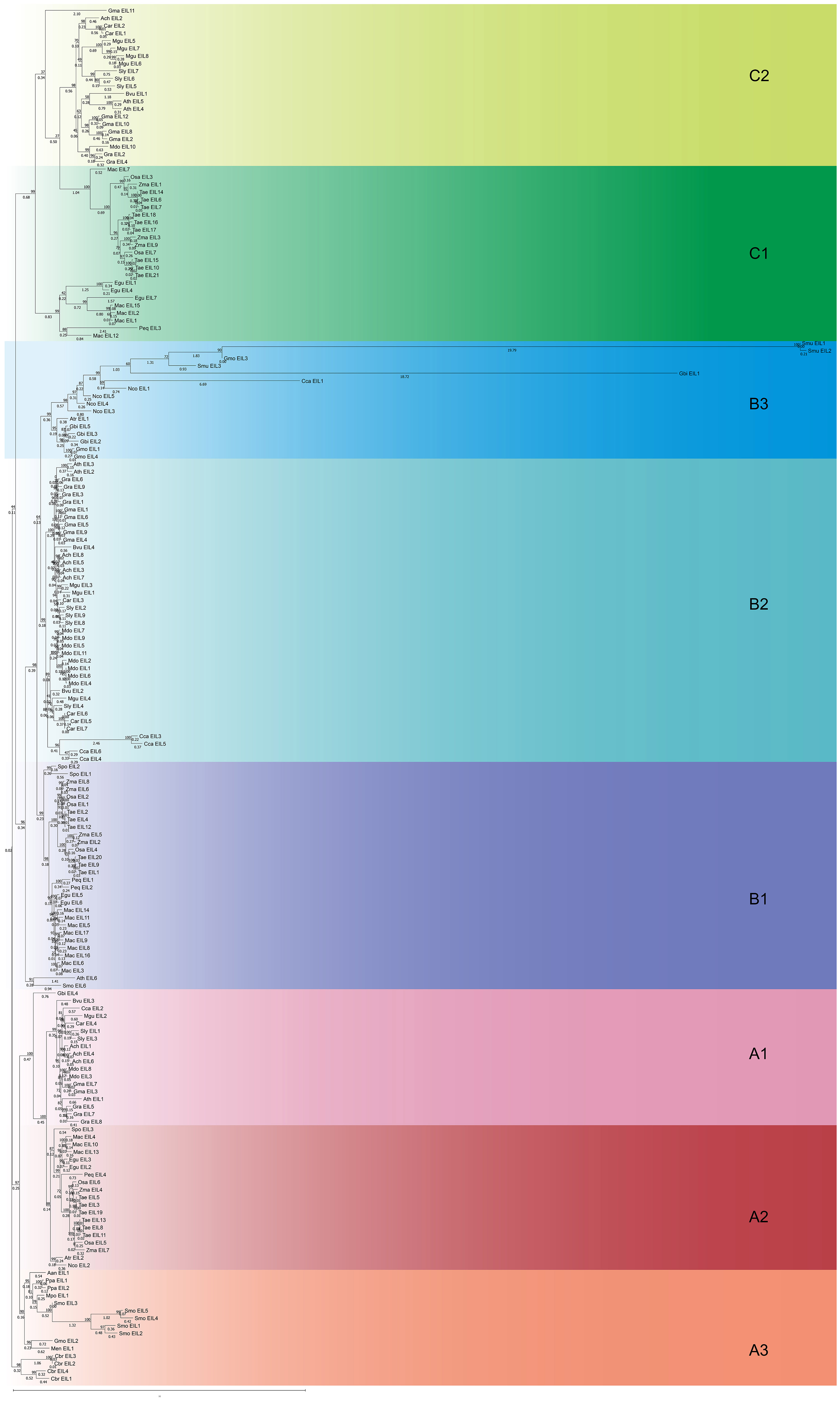

Supplement: Supplementary Figure S1 — Phylogenetic analysis of 182 EIL proteins from 28 species. The phylogenetic tree of all sequences was constructed using IQ-TREE 2 by the Maximum Likelihood (ML) method. [file Data_Sheet_1.ZIP › Figure S2.jpg]

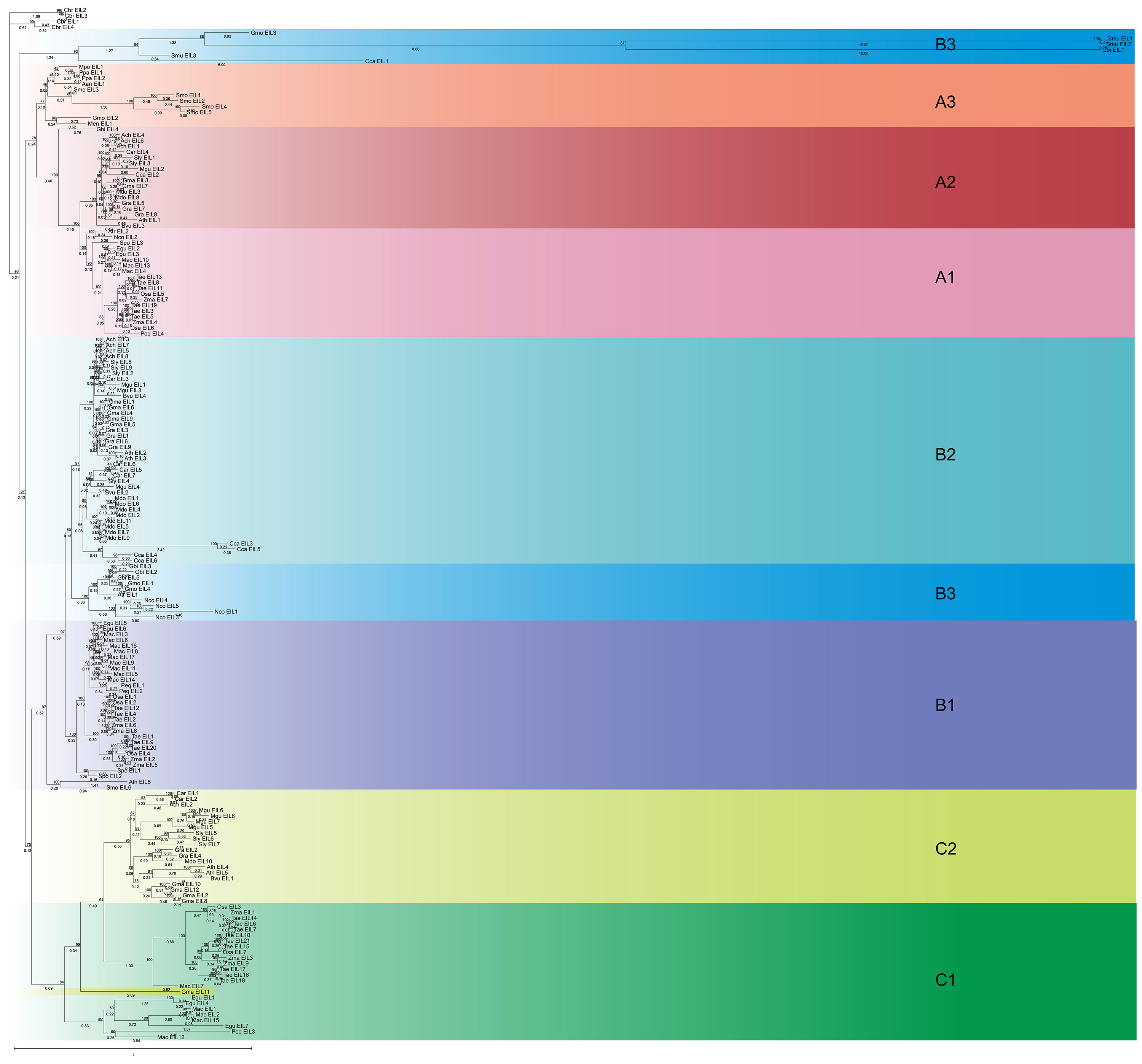

Supplement: Supplementary Figure S1 — Phylogenetic analysis of 182 EIL proteins from 28 species. The phylogenetic tree of all sequences was constructed using IQ-TREE 2 by the Maximum Likelihood (ML) method. [file Data_Sheet_1.ZIP › Figure S3.jpg]

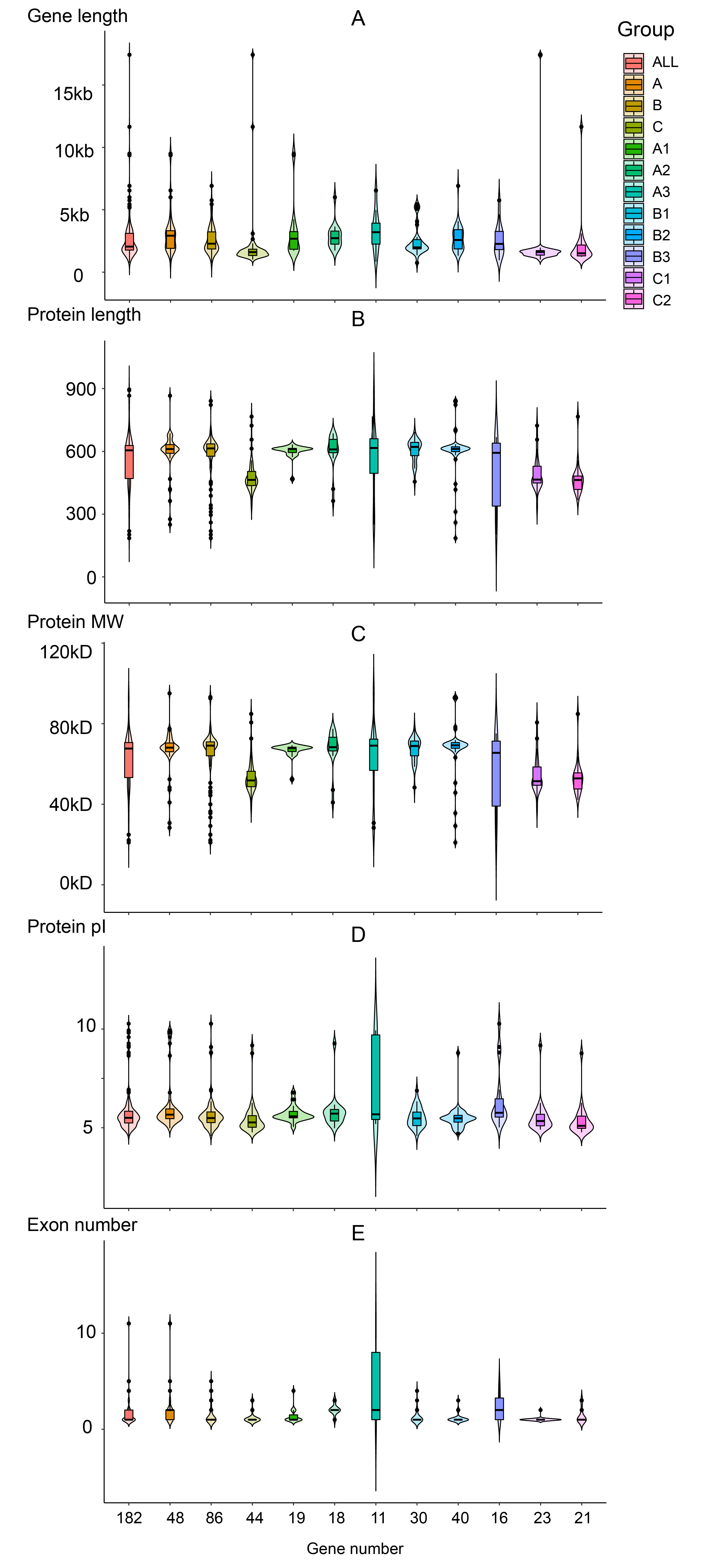

Supplement: Supplementary Figure S1 — Phylogenetic analysis of 182 EIL proteins from 28 species. The phylogenetic tree of all sequences was constructed using IQ-TREE 2 by the Maximum Likelihood (ML) method. [file Data_Sheet_1.ZIP › Figure S4.jpg]

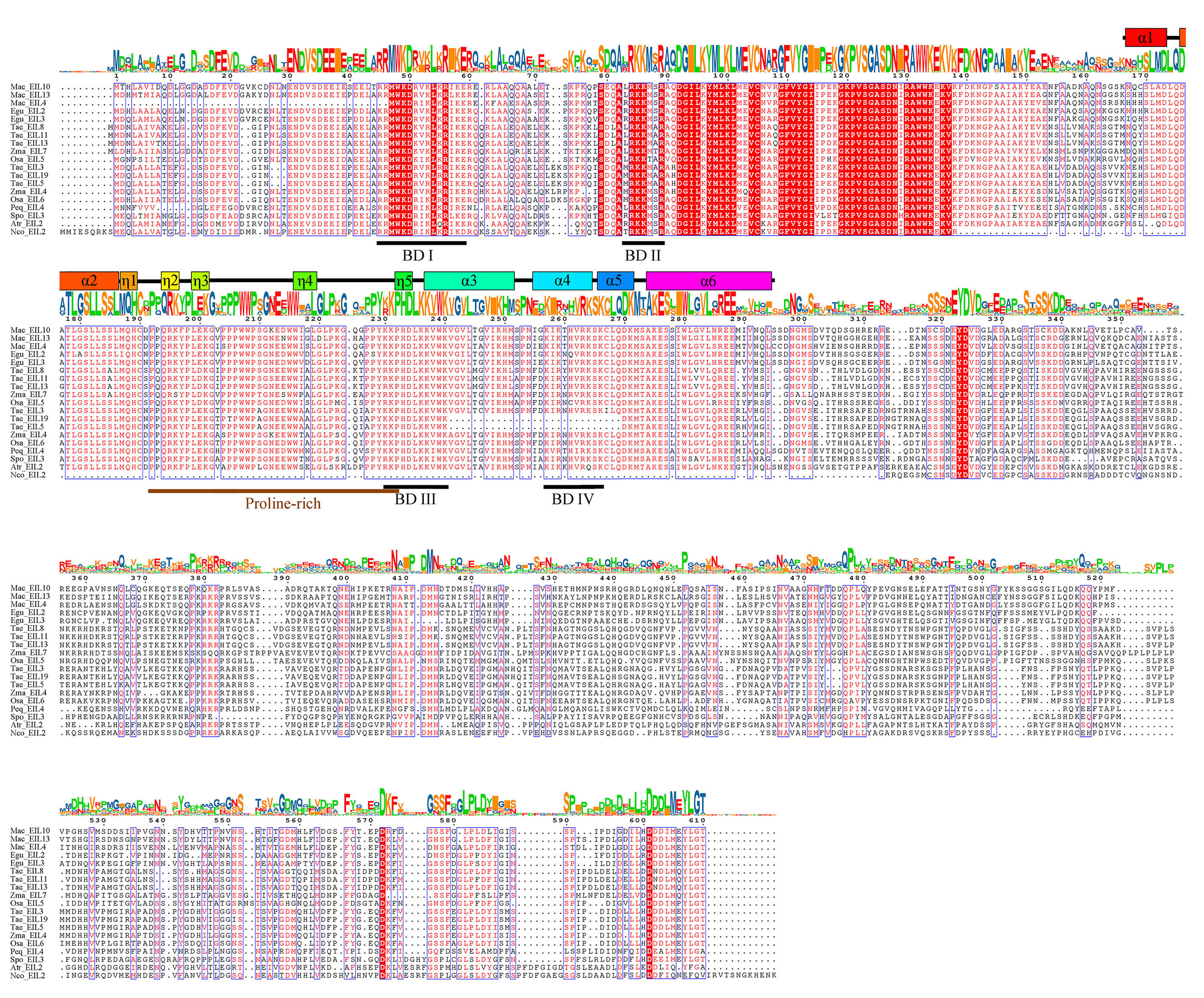

Supplement: Supplementary Figure S1 — Phylogenetic analysis of 182 EIL proteins from 28 species. The phylogenetic tree of all sequences was constructed using IQ-TREE 2 by the Maximum Likelihood (ML) method. [file Data_Sheet_1.ZIP › Figure S5.jpg]

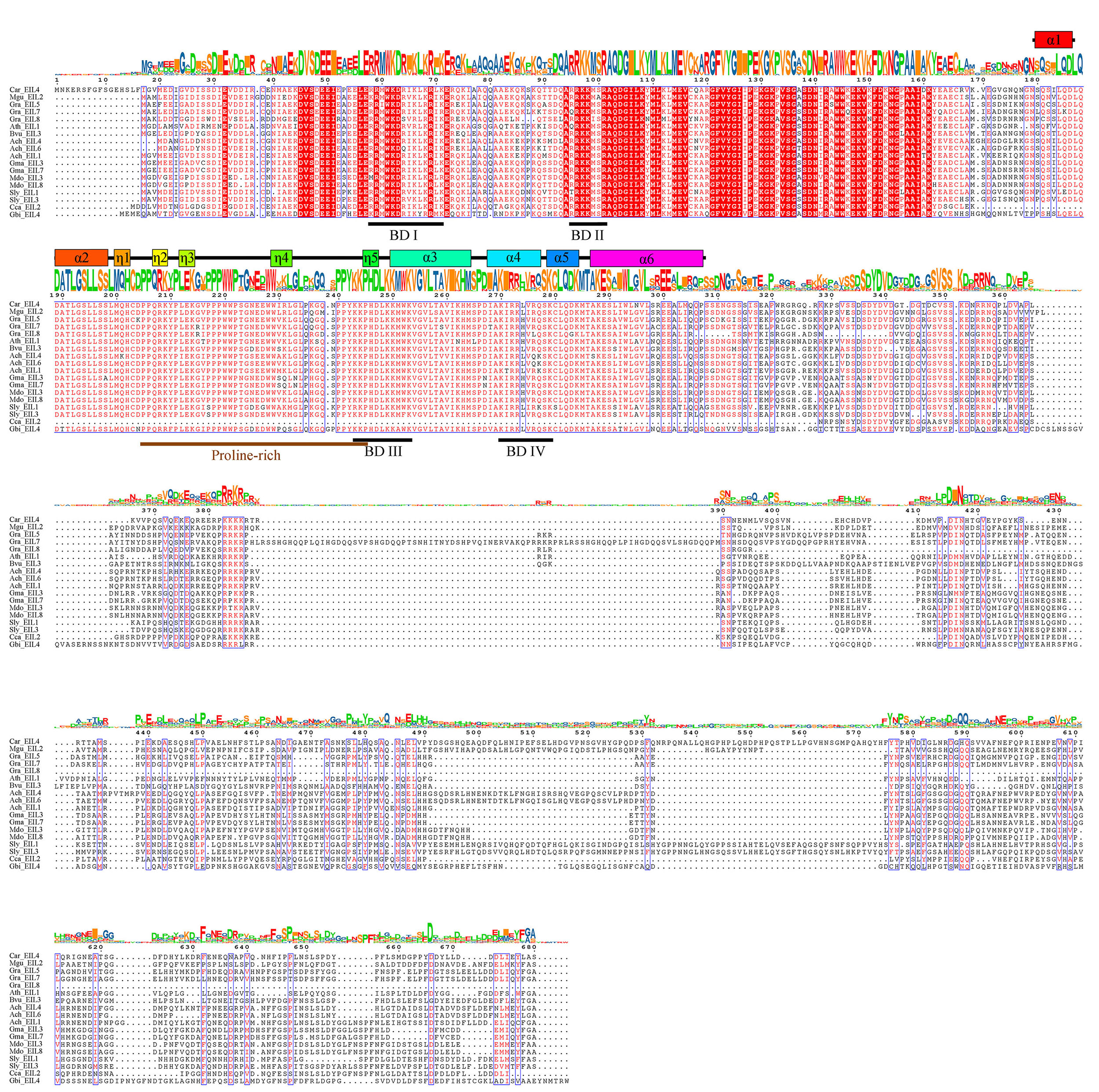

Supplement: Supplementary Figure S1 — Phylogenetic analysis of 182 EIL proteins from 28 species. The phylogenetic tree of all sequences was constructed using IQ-TREE 2 by the Maximum Likelihood (ML) method. [file Data_Sheet_1.ZIP › Figure S6.jpg]

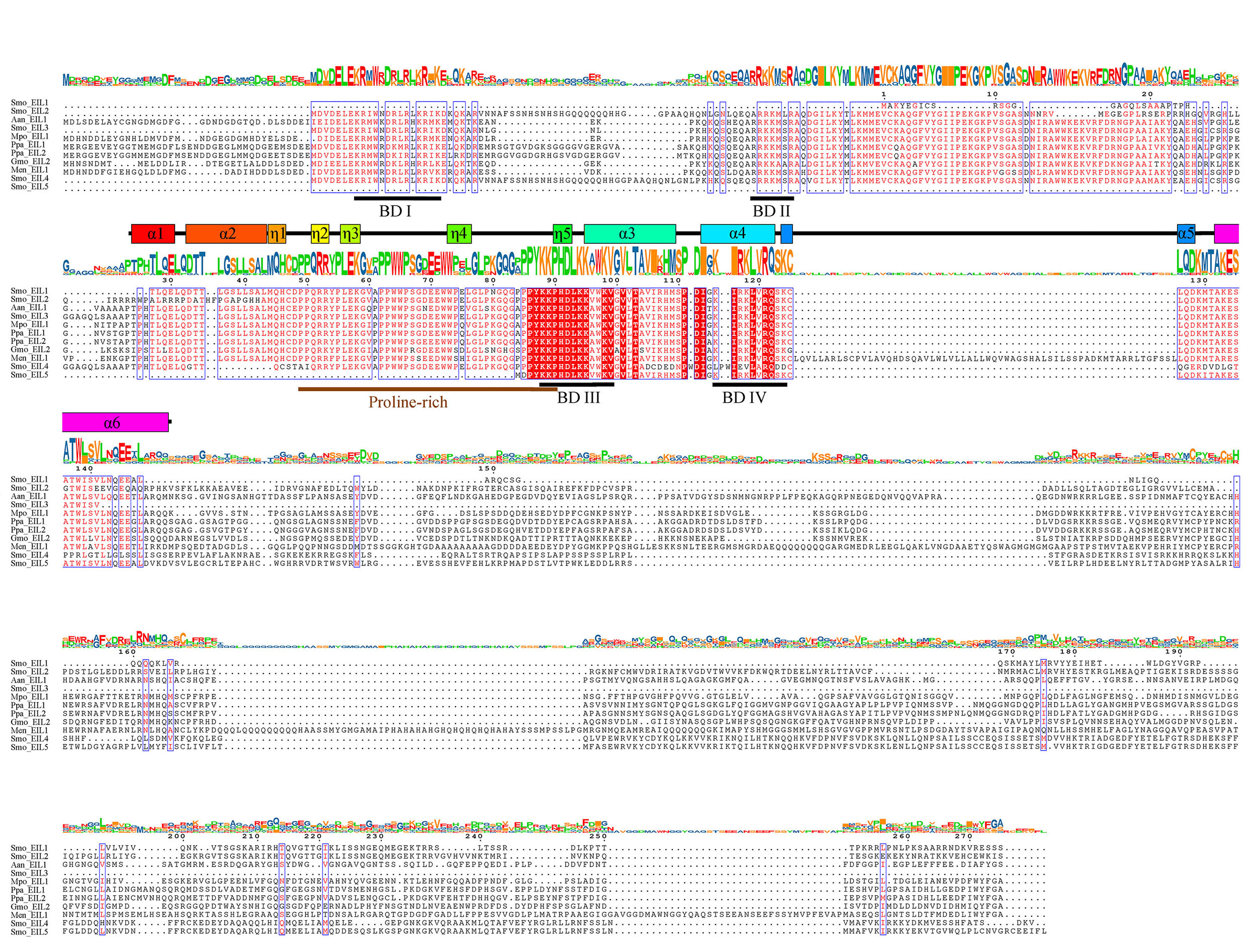

Supplement: Supplementary Figure S1 — Phylogenetic analysis of 182 EIL proteins from 28 species. The phylogenetic tree of all sequences was constructed using IQ-TREE 2 by the Maximum Likelihood (ML) method. [file Data_Sheet_1.ZIP › Figure S7.jpg]

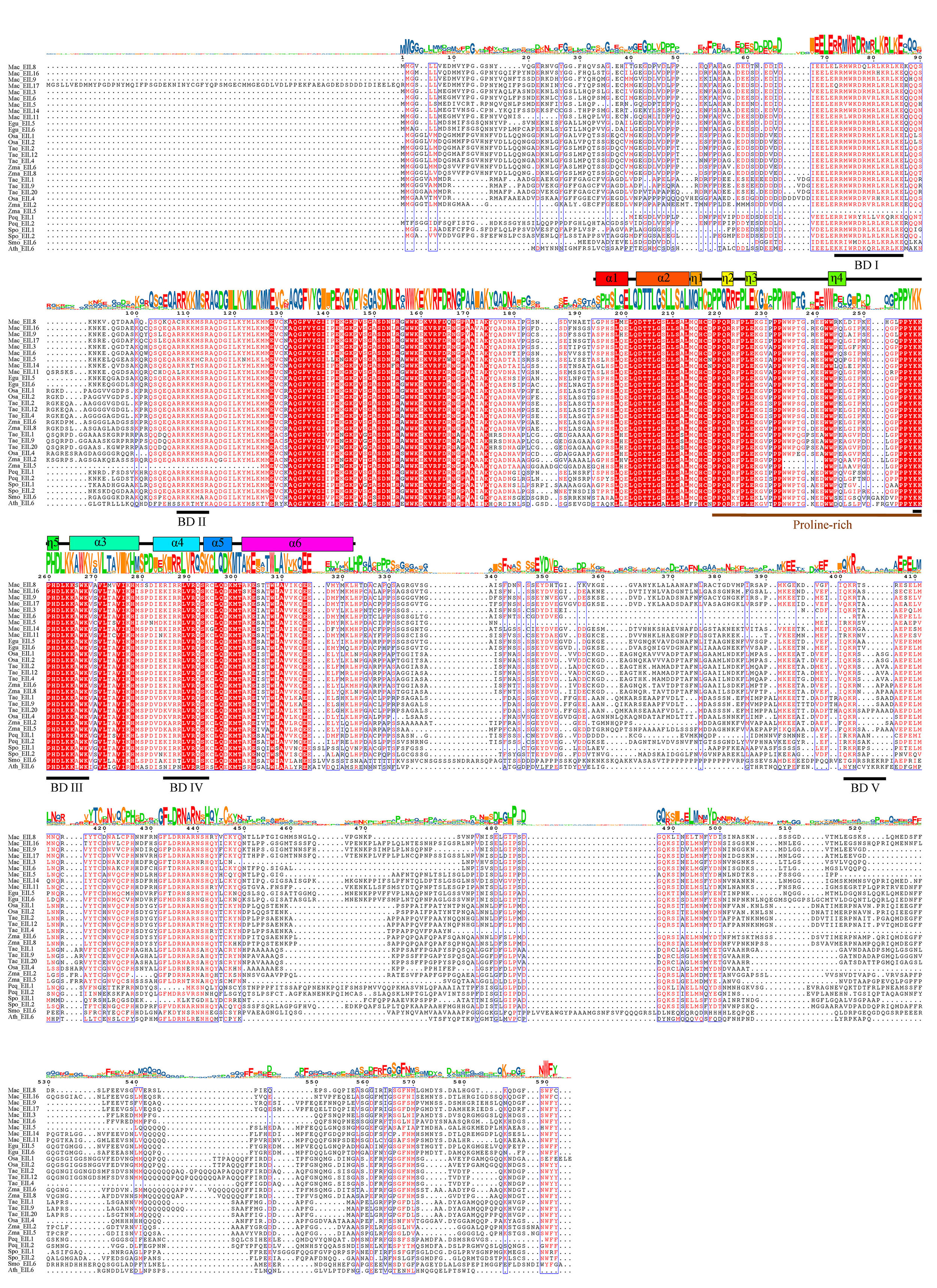

Supplement: Supplementary Figure S1 — Phylogenetic analysis of 182 EIL proteins from 28 species. The phylogenetic tree of all sequences was constructed using IQ-TREE 2 by the Maximum Likelihood (ML) method. [file Data_Sheet_1.ZIP › Figure S8.jpg]

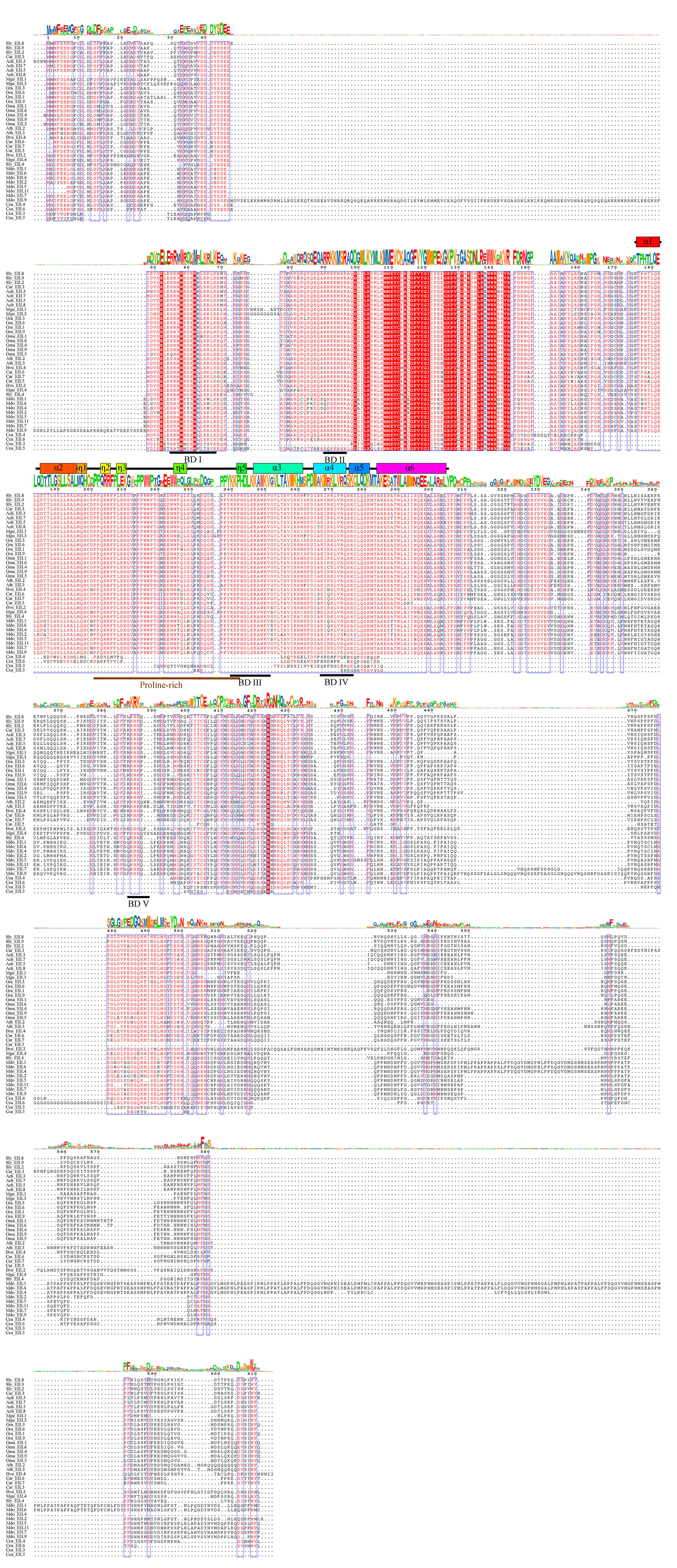

Supplement: Supplementary Figure S1 — Phylogenetic analysis of 182 EIL proteins from 28 species. The phylogenetic tree of all sequences was constructed using IQ-TREE 2 by the Maximum Likelihood (ML) method. [file Data_Sheet_1.ZIP › Figure S9.jpg]

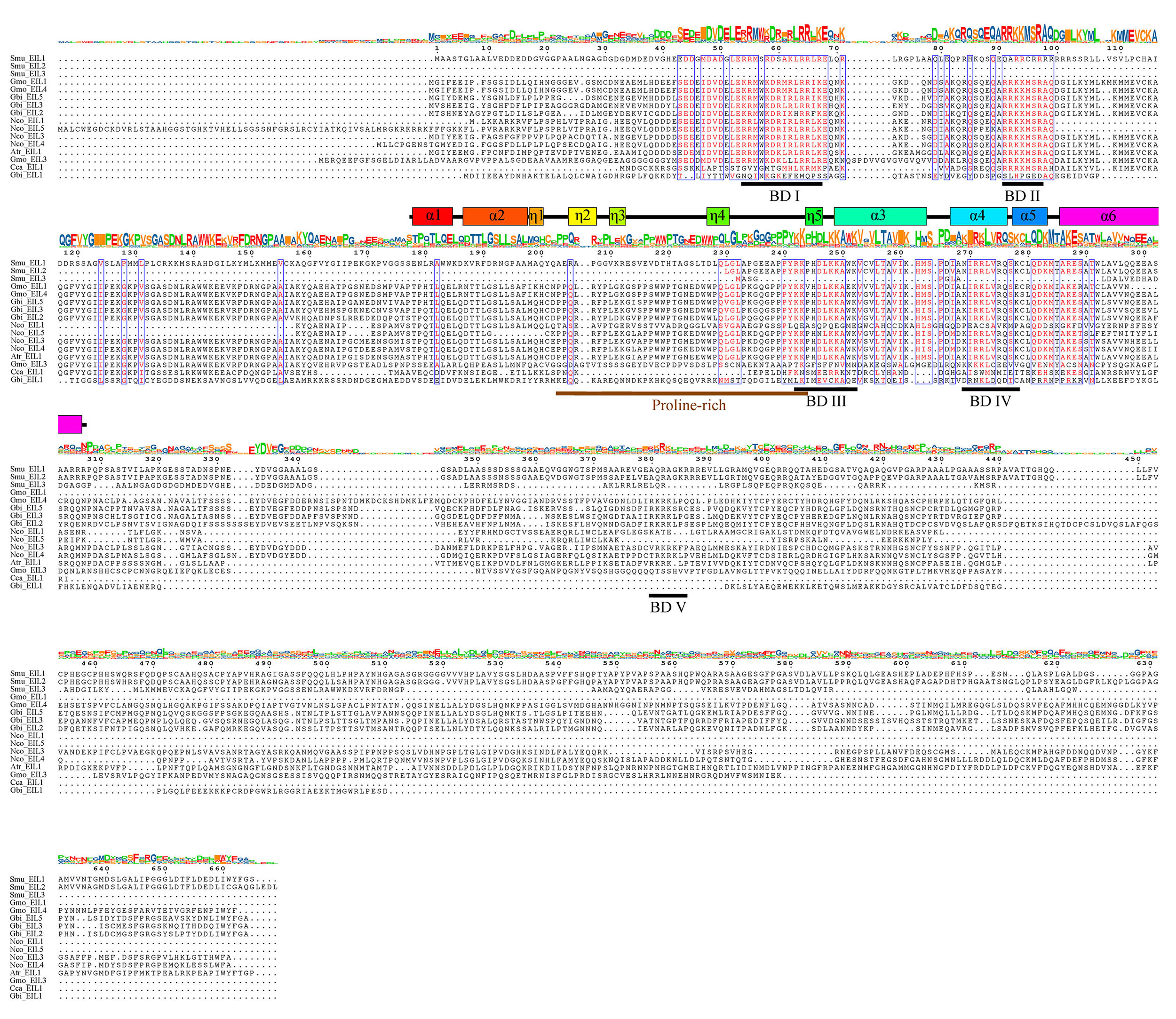

Supplement: Supplementary Figure S1 — Phylogenetic analysis of 182 EIL proteins from 28 species. The phylogenetic tree of all sequences was constructed using IQ-TREE 2 by the Maximum Likelihood (ML) method. [file Data_Sheet_1.ZIP › Figure S10.jpg]

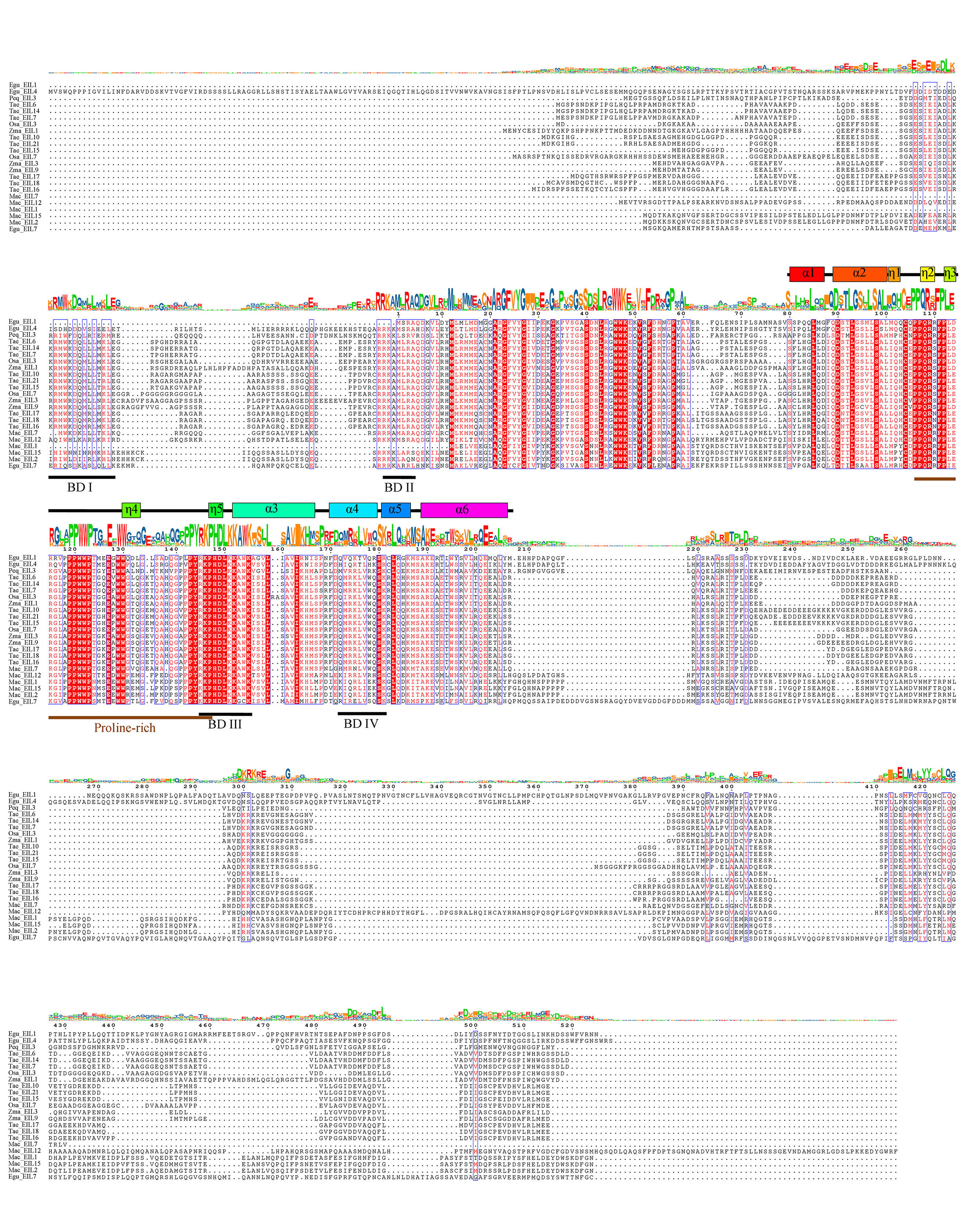

Supplement: Supplementary Figure S1 — Phylogenetic analysis of 182 EIL proteins from 28 species. The phylogenetic tree of all sequences was constructed using IQ-TREE 2 by the Maximum Likelihood (ML) method. [file Data_Sheet_1.ZIP › Figure S11.jpg]

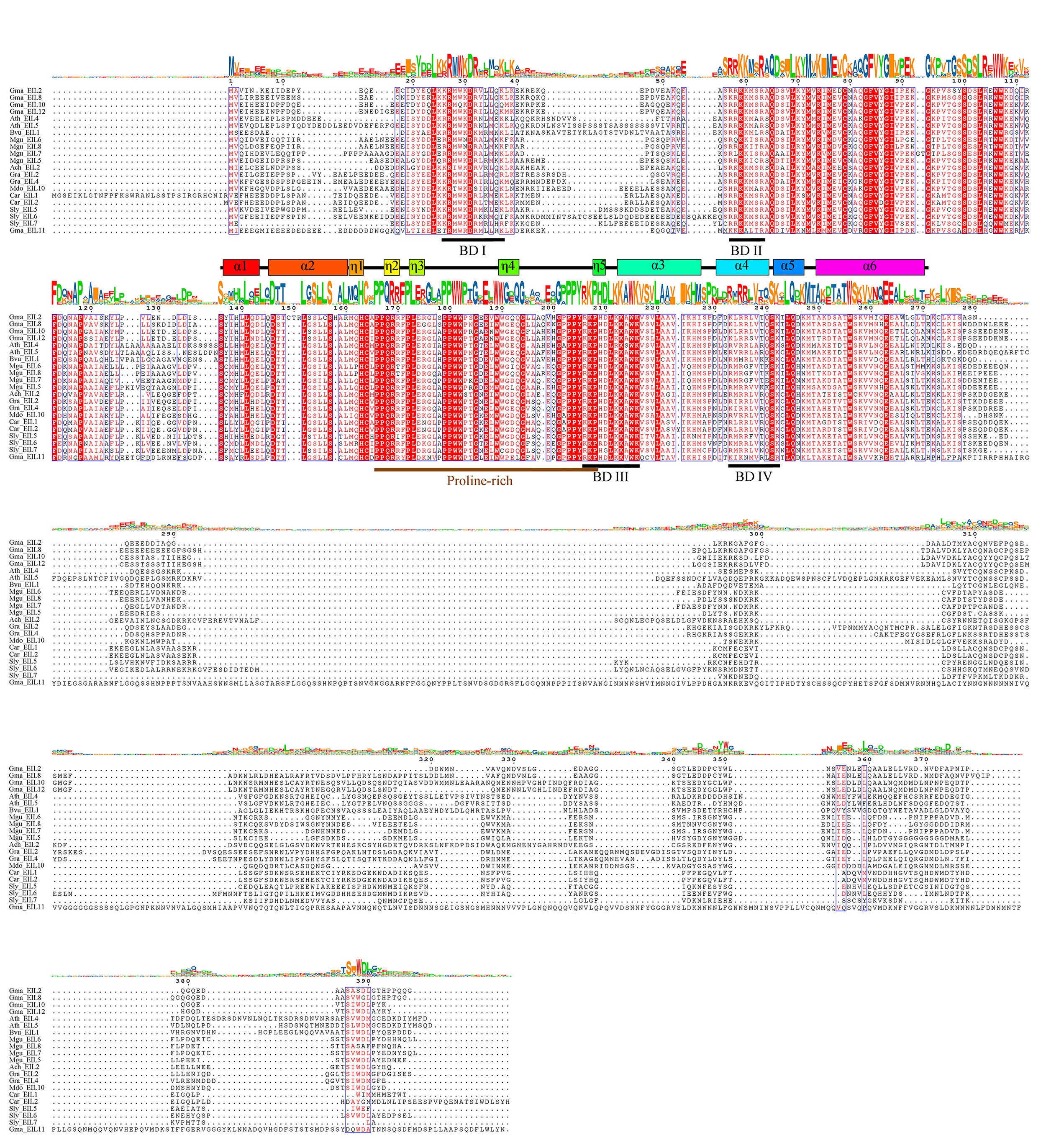

Supplement: Supplementary Figure S1 — Phylogenetic analysis of 182 EIL proteins from 28 species. The phylogenetic tree of all sequences was constructed using IQ-TREE 2 by the Maximum Likelihood (ML) method. [file Data_Sheet_1.ZIP › Figure S12.jpg]
